# Before Sepsis Strikes: Artificial Intelligence for Early Prediction of Postoperative Sepsis and Sepsis-Related Complications After Major Abdominal Surgery—A Systematic Review

**DOI:** 10.3390/medsci14060685

**Published:** 2026-10-19

**Authors:** Wasim Iyad Ibrahim Alghoul, Ibrahim Alabid, Malak Moones Abedi, Mohamedanas Mohamedfaruk Patni, Biji Thomas George, Subhranshu Sekhar Kar, Rajani Dube, Wed Burhan Jameel Al-Shammari, Mohamed Osman Elhag Osman, Riza Jazlin Neyyappadath, Subhanka Nagabhatla, Aya Sabir Salah Ahmed

**Affiliations:** 1 RAK College of Medical Sciences, RAK Medical and Health Sciences University, Ras Al Khaimah 11172, United Arab Emirates; wasim.21901111@rakmhsu.ac.ae (W.I.I.A.); malak.abedi0320@gmail.com (M.M.A.); wed.23901093@rakmhsu.ac.ae (W.B.J.A.-S.); mohamed.o.elhag@gmail.com (M.O.E.O.); riza.21901063@rakmhsu.ac.ae (R.J.N.); subhankasharma96@gmail.com (S.N.); 2 Department of Community Medicine, RAK College of Medical Sciences, RAK Medical and Health Sciences University, Ras Al Khaimah 11172, United Arab Emirates; 3 Department of Surgery, RAK Medical and Health Sciences University, Ras Al Khaimah 11172, United Arab Emirates; biji@rakmhsu.ac.ae; 4 Department of Paediatrics and Neonatology, RAK College of Medical Sciences, RAK Medical and Health Sciences University, Ras Al Khaimah 11172, United Arab Emirates; subhranshu.kar@rakmhsu.ac.ae; 5 Department of Obstetrics and Gynaecology, RAK College of Medical Sciences, RAK Medical and Health Sciences University, Ras Al Khaimah 11172, United Arab Emirates; rajani.dube@rakmhsu.ac.ae; 6 College of Medicine, Ajman University, Ajman 346, United Arab Emirates; 202510771@ajmanuni.ac.ae

**Keywords:** artificial intelligence, machine learning, postoperative sepsis, surgical site infection, anastomotic leakage, postoperative pancreatic fistula, abdominal surgery, risk prediction

## Abstract

Background: Postoperative sepsis and related infectious complications remain major causes of morbidity after abdominal surgery. Artificial intelligence (AI) and machine learning (ML) models may support earlier risk stratification, but their generalizability and clinical readiness remain uncertain. Methods: We systematically searched PubMed, Scopus, and CENTRAL from inception to 10 August 2026 for studies developing, validating, or evaluating AI/ML models for postoperative sepsis, postoperative infection/surgical-site infection (SSI), anastomotic leakage, or postoperative pancreatic fistula (POPF) after major abdominal or general surgery. SSI, anastomotic leakage, and POPF were analyzed as distinct postoperative endpoints and were not treated as proxy definitions of clinical sepsis. Study selection and data extraction were performed independently by two reviewers, and risk of bias and applicability were assessed using PROBAST + AI. Owing to substantial clinical and methodological heterogeneity, results were synthesized narratively. The review was registered in PROSPERO (CRD420261492111). Results: Forty-five studies were included: 11 addressed sepsis-related outcomes, including six studies with a primary sepsis-related modeled endpoint whose definitions ranged from consensus Sepsis-3 criteria to coded or SIRS-based definitions and a qSOFA-based proxy; 19 evaluated postoperative infection/SSI; eight anastomotic leakage; five POPF; and two broader postoperative complications. Performance varied according to outcome, surgical population, prediction timing, predictor availability, and validation strategy. Fifteen of 45 studies provided strict temporal, geographic/external-center, or independent-investigator evaluation of the principal model, with variable preservation of performance outside development settings. More complex ML methods did not reliably outperform conventional regression. Explicit calibration analysis was reported in 9/45 studies, decision-curve or net-benefit analysis in 7/45, and no study prospectively evaluated the impact of model-guided care on clinical outcomes. Certainty of evidence was very low for primary postoperative sepsis/septic-shock prediction, low for postoperative infection/SSI, and very low for anastomotic leakage and POPF. Conclusions: Although several AI/ML models showed promising predictive performance, the available evidence remains insufficient to support routine use of AI/ML prediction models across major abdominal surgical populations. Future studies should prioritize clearly defined clinical use cases, multicenter external validation, calibration, prospective workflow evaluation, and demonstration of actionable clinical benefit before routine implementation.

## 1. Introduction

Postoperative sepsis remains a major complication of abdominal surgery, where earlier identification of high-risk patients may create opportunities for intensified surveillance, investigation, and intervention before overt deterioration. Recent studies have therefore focused on perioperative prediction across abdominal surgery, intestinal-obstruction surgery, and acute surgical populations [1,2,3].

Postoperative sepsis was considered separately from surgical-site or organ-space infection, anastomotic leakage, and postoperative pancreatic fistula, which were treated as distinct outcomes rather than surrogate definitions of sepsis. Machine learning models have been developed for each, including SSI after major abdominal and gastrointestinal surgery, colorectal anastomotic leakage, and pancreatic fistula after pancreaticoduodenectomy [4,5,6,7]. Their inclusion preserves clinically relevant evidence on complications that may precede infectious deterioration without conflating them with primary sepsis-related prediction.

AI and machine learning are well suited to perioperative risk because they can integrate demographic, comorbidity, laboratory, operative, physiological, imaging, and electronic-health-record data [1,7,8,9,10]. Prediction timing is equally important: preoperative models support baseline stratification and prevention; perioperative models can inform disposition and monitoring; and early postoperative or dynamic models may detect evolving complications. Model value therefore depends not only on discrimination but also on whether predictions are timely enough to influence care.

High development performance does not establish clinical readiness. Surgical prediction models remain vulnerable to overfitting, case-mix and institutional differences, missing or inconsistently collected variables, and performance drift outside the development setting; temporal and external validation are therefore critical [5,6,9,10]. The literature also varies substantially in algorithms, predictors, outcomes, prediction windows, validation strategies, calibration, utility, and implementation. Previous systematic reviews have examined artificial intelligence for postoperative complications after major abdominal surgery, compared machine learning with conventional regression across gastrointestinal surgical outcomes, or focused specifically on postoperative surgical-site infection prediction [11,12,13]. These reviews identified promising predictive performance but recurrent limitations in external validation, methodological quality, and reporting. The present review extends this literature by integrating primary sepsis-related prediction with postoperative infection/SSI, anastomotic leakage, and POPF; distinguishing future-event prediction, evolving-current detection, and retrospective case ascertainment; auditing predictor timing and outcome definitions; applying PROBAST + AI; and evaluating validation, calibration, decision-analytic utility, and prospective implementation across clinically distinct prediction tasks. Accordingly, this review evaluates not only how well models discriminate, but what they predict, when the prediction becomes available, how independently performance was evaluated, and whether the evidence is sufficiently mature for clinical translation.

## 2. Materials and Methods

### 2.1. Study Design, Reporting, and Registration

This systematic review was reported in accordance with the Preferred Reporting Items for Systematic Reviews and Meta-Analyses (PRISMA) 2020 statement [14]. The literature search was completed on 10 August 2026. While the systematic review was ongoing, protocol version 1.0 was finalized on 30 August 2026 and submitted to PROSPERO before formal data extraction, risk-of-bias assessment, and evidence synthesis were undertaken. At that time, database searching and study screening had already been completed. The protocol was subsequently registered in PROSPERO on 1 September 2026 (CRD420261492111). The protocol documented the eligibility criteria, information sources, search methods, study-selection procedures, data-extraction methods, risk-of-bias approach, and planned evidence-synthesis methods.

### 2.2. Eligibility Criteria

Original human studies were eligible if they developed, validated, updated, or evaluated an artificial intelligence or machine learning (AI/ML) model for prediction or early detection of postoperative sepsis or a prespecified related complication after major abdominal or general surgery. For this review, major abdominal/general surgery comprised gastrointestinal, colorectal, gastric, hepatobiliary, pancreatic, bariatric, abdominal-transplant, emergency-abdominal, and trauma-related abdominal procedures, using elective or emergency and open or minimally invasive approaches. In accordance with the prespecified protocol, mixed surgical cohorts remained eligible when abdominal/general-surgical patients were represented. For synthesis, studies in which the abdominal/general-surgical population or its performance estimates were separately identifiable were treated as direct evidence for the target population; mixed multispecialty studies without separately extractable abdominal-surgical performance were retained as indirect contextual evidence.

Eligible approaches included machine learning, deep learning, neural networks, random forests, support-vector machines, gradient boosting, XGBoost, CatBoost, ensembles, Bayesian networks, natural-language processing, and other data-driven predictive models using preoperative, intraoperative, perioperative, or early postoperative information. At least one eligible AI/ML or data-driven modeling approach beyond conventional unpenalized regression alone was required for study inclusion. Conventional regression models were retained when reported as comparators within otherwise eligible studies. A formal comparator was not required.

For synthesis, each model evaluation was additionally classified according to its intended clinical task as: (1) prognostic prediction of a future postoperative complication; (2) early detection or monitoring of an evolving or currently developing postoperative complication; or (3) retrospective case ascertainment or surveillance. Classification was based on the intended use of the model and the temporal ordering between predictor availability and outcome ascertainment rather than solely on labels such as preoperative, perioperative, or postoperative. Postoperative predictors were not automatically considered evidence of information leakage; instead, the latest permissible predictor time, outcome-assessment window, and any reported lead time were examined to determine whether the model provided pre-event prediction, near-event detection, or retrospective case identification.

Outcomes were classified as: (1) postoperative sepsis/septic shock; (2) postoperative infection, SSI, or deep/organ-space infection; (3) anastomotic leakage; (4) postoperative pancreatic fistula; and (5) other sepsis-related or major abdominal postoperative complications. Leakage and fistula were analyzed as distinct complications, not as equivalent definitions of sepsis; broader composite models containing infectious and noninfectious outcomes were retained for contextual synthesis but analyzed separately from primary sepsis-related outcomes. Eligible designs included retrospective or prospective cohorts, registries, EHR analyses, multicenter studies, and prediction-model development/validation studies. Reviews, meta-analyses, editorials, commentaries, protocols, case reports, animal/laboratory studies, conference abstracts without sufficient data, and AI applications unrelated to postoperative risk prediction were excluded.

### 2.3. Information Sources and Search Strategy

PubMed, Scopus, and the Cochrane Central Register of Controlled Trials (CENTRAL) were searched from database inception through 10 August 2026 without language or starting-date restrictions. The strategy covered three domains: AI/ML, postoperative sepsis and related complications, and abdominal/general surgery. PubMed and Scopus combined all three; CENTRAL intentionally used the broader AI/ML-plus-outcome strategy to maximize sensitivity, with surgical eligibility applied during screening. Controlled vocabulary and free-text terms covered major AI methods, sepsis/infection/SSI, anastomotic leakage, pancreatic fistula, postoperative complications, and relevant abdominal-surgical procedures.

The searches retrieved 1029 PubMed, 668 Scopus, and 208 CENTRAL records (1905 before deduplication). Reference lists of included studies and relevant reviews were checked and forward citation searching was performed. Full reproducible strategies, field restrictions, and filters are provided in Supplementary Table S1.

### 2.4. Study Selection

All identified records were imported into EndNote 2025.4 for reference management and duplicate removal before screening. Two reviewers independently screened titles and abstracts against the prespecified eligibility criteria. Records considered potentially relevant by either reviewer proceeded to full-text assessment.

**Figure 1:**
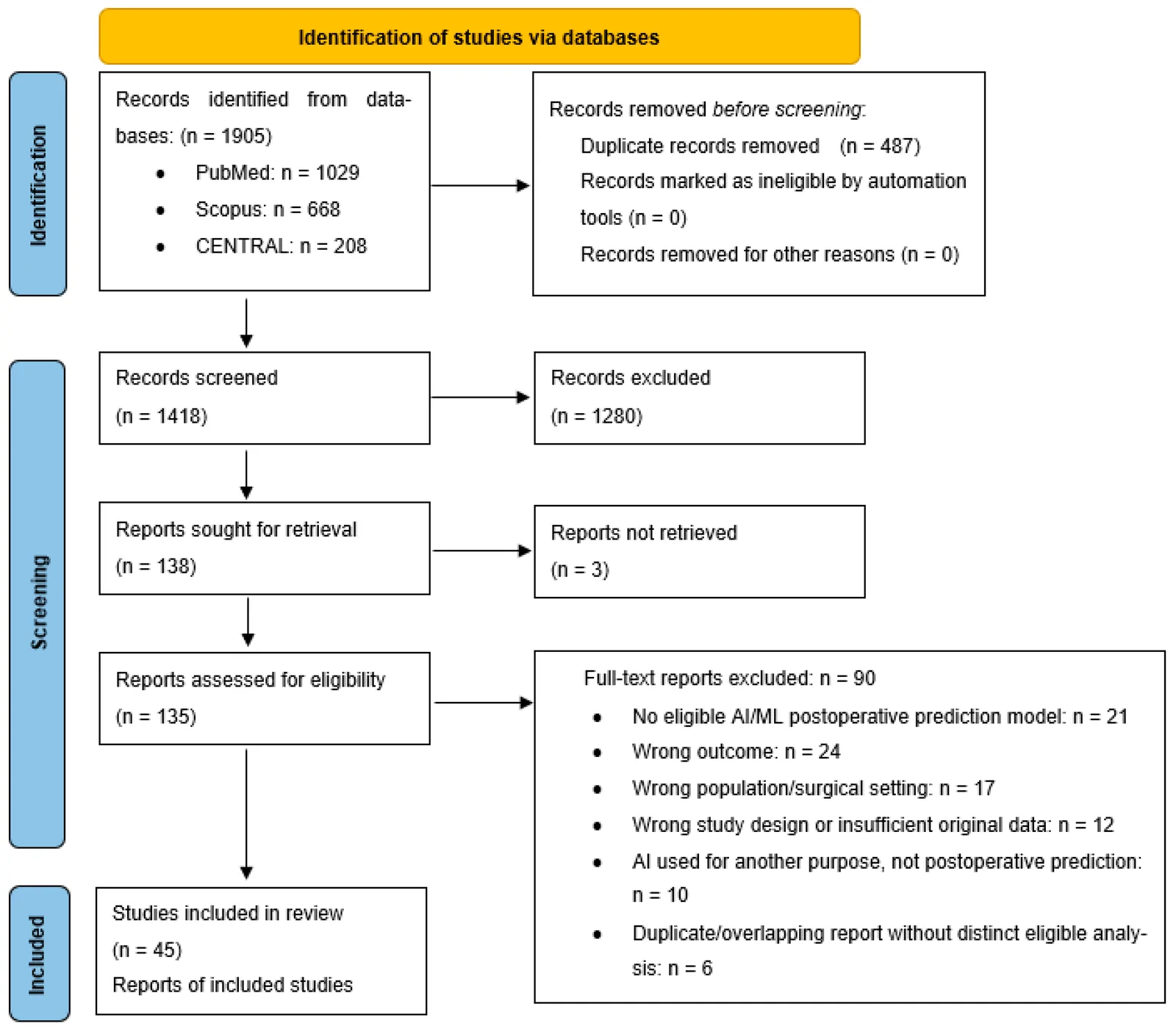
PRISMA 2020 flow diagram illustrating the identification, screening, eligibility assessment, and final inclusion of studies evaluating artificial intelligence and machine learning models for postoperative sepsis and related complications after major abdominal surgery.

Full-text reports were independently evaluated by two reviewers. Disagreements were resolved through discussion and consensus, with adjudication by a third reviewer when necessary. Reasons for exclusion were recorded for all reports excluded at the full-text stage. Multiple reports originating from the same underlying cohort were linked and treated as a single study where appropriate, while retaining distinct analyses when they evaluated materially different models, outcomes, or validation cohorts. The complete study-selection process, including record identification, duplicate removal, screening, full-text assessment, exclusions, and final study inclusion, is summarized in the PRISMA 2020 flow diagram (Figure 1).

### 2.5. Data Extraction and Data Items

Two reviewers independently extracted data using a standardized form; disagreements were resolved by consensus and, when required, a third reviewer. Data items and prediction-model characteristics were informed by the CHARMS framework [15].

Extracted study characteristics included author, year, country, setting, centers, design, data source, recruitment period, surgical population, sample size, outcome events, and prevalence. Model characteristics included algorithms, candidate and final predictors, feature selection/dimensionality reduction, missing-data and class-imbalance handling, hyperparameter optimization, training/testing procedures, and model-selection strategy. Predictors were grouped as demographic, comorbidity-related, clinical, laboratory, inflammatory, physiological, imaging-derived, intraoperative, or postoperative.

Prediction timing was classified as preoperative, intraoperative/perioperative, early postoperative, or dynamic/real-time, with prediction horizons recorded when reported. Outcome definitions were audited at the study level, including the terminology and diagnostic criteria used by the primary authors, method of outcome ascertainment, timing relative to surgery, and follow-up interval. For sepsis-related outcomes, extraction additionally recorded whether infection and organ dysfunction were required, whether Sepsis-3 or earlier sepsis criteria were used, whether septic shock was included, whether the outcome was based on administrative coding or a score-based proxy such as qSOFA, and whether pre-existing sepsis was excluded. For SSI, anastomotic leakage, and postoperative pancreatic fistula, study-specific diagnostic definitions, subtypes or severity grades, ascertainment methods, and follow-up intervals were recorded. Score-based proxies, administrative definitions, and consensus-defined or clinically adjudicated outcomes were distinguished rather than treated as interchangeable manifestations of the same endpoint.

Additional extraction covered calibration, explainability, decision-curve analysis/net benefit, decision-support tools, prospective implementation, funding, and conflicts of interest. When multiple horizons, models, or metrics were reported, clinically relevant data were retained, while the principal synthesis prioritized the authors’ final/prespecified model and the most independent validation dataset.

### 2.6. Model Performance Measures

Discrimination was primarily assessed using AUROC/AUC or c-statistic with 95% confidence intervals where reported; AUPRC was also extracted, particularly for imbalanced outcomes. Classification metrics included sensitivity, specificity, positive and negative predictive values, accuracy, precision, recall, and F1-score, prioritizing the prespecified or principal clinically relevant threshold.

Calibration measures included calibration plots, calibration-in-the-large/intercept, calibration slope, observed-to-expected ratios, and other reported calibration statistics. The Brier score was extracted separately as a measure of overall probabilistic prediction error. Goodness-of-fit testing alone was not considered sufficient evidence of calibration when more informative measures were available. Clinical usefulness was summarized using decision-curve analysis, net benefit, clinical-impact curves, or related measures, while explainability methods such as feature importance and SHAP were considered separately from predictive performance.

Development-set discrimination was not treated as equivalent to externally validated performance; all performance estimates were interpreted in the context of their validation method.

### 2.7. Risk of Bias, Model-Development Quality, and Applicability Assessment

Methodological quality, risk of bias, and applicability were assessed independently by two reviewers using PROBAST + AI, the updated Prediction model Risk Of Bias Assessment Tool for prediction models developed using regression or artificial-intelligence/machine learning methods [16]. PROBAST + AI distinguishes assessment of model development from assessment of model evaluation. Model-development studies were assessed for methodological quality, whereas evaluations of model performance were assessed for risk of bias.

For both components, four domains were considered: (1) participants and data sources, (2) predictors, (3) outcome, and (4) analysis. Applicability to the present review question was assessed separately for the participants/data sources, predictors, and outcome domains. Signaling questions were answered as “yes,” “probably yes,” “probably no,” “no,” “no information,” or “not applicable,” and domain-level judgments were subsequently assigned as low, unclear, or high concern as appropriate.

The assessment specifically considered methodological issues relevant to AI/ML prediction research, including participant selection, predictor availability at the intended time of model use, temporal leakage, repeated-patient overlap between development and evaluation datasets, preprocessing and feature selection outside resampling procedures, separation of hyperparameter tuning from performance evaluation, handling of missing data and class imbalance, recalibration following resampling or model updating, case–control sampling, sample size and number of outcome events, and independence of development and validation datasets. A negative or probably negative signaling response was not automatically interpreted as high risk; domain judgments reflected the likely mechanism and potential direction of bias.

Where several algorithms were developed and evaluated using the same participants, predictors, outcome definition, preprocessing, and evaluation pipeline, the principal or final model used in the synthesis was assessed, with common methodological judgments considered applicable to comparator models unless their development or evaluation procedures differed materially. For evaluation, the most independent available performance assessment was prioritized, with independent, geographic, or temporal validation preferred over internal holdout or resampling where available. Detailed signaling-question responses, domain-level judgments, overall assessments, applicability judgments, and source locations are provided in Supplementary Table S4.

ROBINS-I had been specified in protocol version 1.0 because the review predominantly included non-randomized observational study designs, and it was initially retained as a general risk-of-bias framework for non-randomized evidence. The protocol itself acknowledged that ROBINS-I was developed for non-randomized studies of interventions rather than specifically for prediction-model development and therefore planned cautious interpretation alongside CHARMS-informed prediction-model-specific methodological features. Following PROSPERO registration, but before the formal risk-of-bias assessment was undertaken, the appraisal approach was amended to PROBAST + AI [16]. This change was made because the included evidence consisted predominantly of prediction-model development and evaluation studies, for which PROBAST + AI provided the more appropriate prediction-model-specific framework for both regression- and AI/ML-based models. The amendment was made before formal risk-of-bias assessment and evidence synthesis and did not alter study eligibility, outcome definitions, or the prespecified clinical outcome groups.

### 2.8. Data Synthesis and Statistical Analysis

Studies were organized by prespecified outcome group—sepsis/septic shock, infection/SSI, anastomotic leakage, pancreatic fistula, and other relevant postoperative complications—and then by surgical population, prediction timing, model type, and validation design. Structured narrative synthesis summarized study/model characteristics, predictors, validation, performance, calibration, utility, and implementation.

Quantitative pooling was prespecified only when at least three studies were clinically and methodologically comparable in outcome and definition, population, prediction horizon, evaluation stage, and metric; sharing a metric alone was insufficient. Development and external-validation estimates were not combined indiscriminately, correlated models from the same cohort were not treated as independent, and the authors’ final or independently validated model was prioritized when multiple algorithms were tested.

Random-effects meta-analysis, with 95% confidence intervals, tau-squared/I-squared heterogeneity estimates and prediction intervals where feasible, and bivariate random-effects or hierarchical summary ROC methods for sufficiently comparable sensitivity/specificity data were prespecified but not undertaken because no subgroup met pooling criteria. Potential heterogeneity was considered by procedure, outcome, prediction timing, model class, design, center structure, and validation type; meta-regression was contingent on sufficient studies.

Because quantitative pooling was not appropriate, sensitivity analyses were performed narratively rather than statistically. The robustness of the principal findings was examined by restricting the evidence to: (1) studies reporting abdominal/general-surgery-specific performance; (2) evaluations representing genuine pre-event prognostic prediction or early warning, excluding retrospective surveillance/case-ascertainment models and evaluations with unresolved temporal-order concerns; (3) primary sepsis-related studies excluding score-based proxy outcomes such as qSOFA; (4) studies or evaluation cohorts using consensus Sepsis-3 definitions; (5) models evaluated in temporal, geographic/external-center, or independent-investigator datasets; (6) studies judged at low overall model-evaluation risk of bias using PROBAST + AI; and (7) studies remaining after exclusion of evaluations judged at high overall risk of bias.

Studies with a primary sepsis-related modeled endpoint were synthesized separately from broader postoperative-complication models and upstream surgical complications. Within the primary sepsis-related evidence, consensus-defined sepsis, administrative or older sepsis criteria, and score-based proxies were not treated as equivalent outcomes. Results of the narrative sensitivity analyses are reported in the Results and detailed in Supplementary Table S5.

### 2.9. Reporting Bias and Certainty of Evidence

The protocol-specified Grading of Recommendations Assessment, Development and Evaluation (GRADE) principles were operationalized for prediction-model evidence to assess certainty separately for four principal clinical outcome domains: primary postoperative sepsis/septic shock, postoperative infection/surgical-site infection, anastomotic leakage, and postoperative pancreatic fistula [17,18].

The target of the certainty assessment was confidence in the reported predictive performance and transportability of models within each outcome domain. Certainty regarding whether model-guided clinical care improves patient outcomes was not assessed, because the included studies predominantly evaluated model development or predictive performance rather than prospective model-guided intervention.

For reproducibility, each outcome domain was assigned an initial certainty of high and could be downgraded by one level for serious concerns or two levels for very serious concerns across five domains: risk of bias, inconsistency, indirectness, imprecision, and publication/reporting bias, to a minimum rating of very low. No upgrading criteria were applied.

Risk of bias was informed primarily by the PROBAST + AI model-evaluation assessments, with particular consideration of studies judged at high overall evaluation risk of bias and recurrent concerns involving participant selection, temporal or data leakage, missing-data handling, feature selection or hyperparameter tuning outside appropriate resampling procedures, and incomplete separation of model development from evaluation. Inconsistency reflected clinically meaningful variation in predictive performance across studies or validation datasets together with heterogeneity in outcome definitions, clinical prediction tasks, prediction timing, model architecture, and validation setting. Indirectness reflected departures from the target review question, including mixed surgical populations without separately extractable abdominal-surgery performance, score-based proxy outcomes, retrospective surveillance or case-ascertainment tasks, and predictor timing inconsistent with the intended pre-event prediction or early-warning setting.

Imprecision was judged from the amount and precision of the most relevant evaluation evidence, particularly the number of outcome events and the width of confidence intervals in temporal, geographic, or independent validation cohorts, rather than from total development-sample size alone. Sparse validation events, wide confidence intervals spanning clinically meaningfully different levels of performance, or absence of uncertainty estimates were considered reasons for concern.

Publication or reporting bias was considered separately. Formal small-study-effect assessment was planned only when at least 10 sufficiently comparable studies were available; because no outcome group met this requirement for quantitative synthesis, funnel plots and regression-based tests were not performed. Publication bias was therefore classified as not formally assessable rather than assumed to be absent.

External validation and applicability were not treated as additional independent GRADE domains, thereby avoiding double counting. Instead, relevant concerns were incorporated within risk of bias, inconsistency, or indirectness as appropriate. Broader postoperative-complication models containing heterogeneous infectious and noninfectious outcomes were retained for contextual synthesis but were not assigned separate certainty ratings within the four principal outcome domains. Final certainty was categorized as high, moderate, low, or very low, with domain-level judgments and their rationale.

## 3. Results

### 3.1. Study Selection and Characteristics

#### 3.1.1. Study Selection

The database search identified 1905 records, including 1029 from PubMed, 668 from Scopus, and 208 from CENTRAL. After removal of 487 duplicate records, 1418 records underwent title and abstract screening. Of these, 1280 records were excluded, and 138 reports were sought for full-text retrieval. Three reports could not be retrieved, leaving 135 full-text reports for eligibility assessment. Following full-text review, 90 reports were excluded for prespecified reasons, and 45 studies were included in the final systematic review. The study-selection process is presented in the PRISMA 2020 flow diagram (Figure 1).

#### 3.1.2. Characteristics of Included Studies

The 45 studies were published from 2014 to 2026; 26/45 (57.8%) appeared from 2022 onward. Sample sizes ranged from 76 to 2,882,526 (median 1961). Evidence was predominantly retrospective and spanned single-center cohorts, multicenter datasets, national registries, EHR repositories, and international collaborations; prospective data collection or prospective/temporal validation was less common [3,5,10,19]. Because some large registries or institutional datasets may overlap, study sample sizes were not summed as unique patients.

Populations covered general/emergency abdominal surgery, appendectomy, intestinal obstruction, colorectal, gastric, esophagogastric, hepatobiliary and pancreatic surgery, bariatric surgery, liver transplantation, and trauma laparotomy [20,21,22,23], with some broader major-surgical cohorts containing relevant abdominal/general-surgery patients [24,25]. Eleven studies addressed sepsis-related outcomes: six evaluated a sepsis-related endpoint as the primary modeled outcome [1,2,3,20,26,27], and five evaluated sepsis or septic shock within broader postoperative-complication models [21,24,25,28,29]. Among the six primary-target studies, outcome definitions were heterogeneous and included consensus Sepsis-3 criteria, administrative/coded or older SIRS-based definitions, and a qSOFA-based proxy; 19 addressed infection/SSI [4,5,8,9,19,22,30,31,32,33,34,35,36,37,38,39,40,41,42]; eight leakage [6,10,23,43,44,45,46,47]; five POPF [7,48,49,50,51]; and two broader postoperative-complication outcomes [52,53]. These categories were analyzed separately.

Prediction timing ranged from preoperative to intraoperative/perioperative, early postoperative, and dynamic longitudinal modeling [19,26,35,42]. These timing categories were not treated as synonymous with the intended clinical task. Based on the temporal relationship between predictor availability and outcome ascertainment, 32 of 45 studies (71.1%) were classified primarily as future-event prognostic prediction, nine (20.0%) as early detection, dynamic monitoring, or sequential postoperative prediction, and four (8.9%) as retrospective surveillance or case ascertainment. Two studies within the future-event prognostic group had specific concerns regarding predictor timing or potential temporal leakage and were therefore interpreted cautiously in the temporal-order assessment.

Surveillance-focused evaluations were retained as contextual evidence but were not interpreted as demonstrating clinically actionable pre-event prediction unless a lead time before outcome identification was demonstrated. This distinction was clinically important because baseline risk stratification, postoperative early warning, and retrospective case finding serve different clinical and operational purposes. Detailed study-level task classification, predictor timing, outcome windows, lead times, and temporal-order assessments are provided in Supplementary Table S2.

Table 1 summarizes study design, setting, population, sample size, outcome, prediction timing, model, and validation strategy.

### 3.2. AI and Machine-Learning Model Characteristics

The studies evaluated logistic regression benchmarks alongside decision trees, random forests, support-vector machines, naive Bayes, neural networks, gradient boosting, XGBoost, CatBoost, ensembles, Bayesian networks, and recurrent architectures; many compared multiple algorithms within one dataset or constructed ensembles [1,8,27,45]. Tree-based/ensemble approaches were common, including Shu et al.’s KNN/SVM ensemble, Chen et al.’s random forest liver-transplant model, SMART gradient boosting, and Ghabisha et al.’s LR/RF/XGBoost/neural-network comparison [1,4,8,27], underscoring that no algorithm was consistently superior across settings.

Inputs ranged from routine demographics, comorbidities, laboratories, ASA class, and operative factors to EHR data, free text, serial wound observations, continuous physiology, longitudinal postoperative measurements, and CT-derived features [7,26,30,36]. High-frequency vital signs were used to forecast sepsis after liver transplantation [26], while pancreatic-fistula models combined clinical data with CT anatomy/body composition [7,48]. Feature selection included clinical selection, recursive feature elimination, regularization, Boruta, filters, and algorithm-derived importance; newer studies increasingly used feature-importance or SHAP explanations [2,7,10,22].

Validation approaches ranged from internal random holdout, cross-validation, and bootstrap resampling to temporal, geographic/external-center, and independent-investigator evaluation. Across the 45 studies, eight reported temporal validation, eight reported geographic or external-center validation, and one represented independent-investigator validation of an existing model; these categories were not mutually exclusive. Two studies additionally included local model updating or recalibration. Overall, 15 of 45 studies (33.3%) provided a strict temporal, geographic/external-center, or independent-investigator evaluation of the principal model used in the synthesis. Detailed validation categories and linked model families are provided in Supplementary Table S5.

Reporting of model performance beyond discrimination was less complete. Explicit calibration analysis was reported in 9 of 45 studies (20.0%), while 12 of 45 (26.7%) provided any calibration-related information. Decision-curve analysis or another net-benefit assessment was reported in 7 of 45 studies (15.6%). None of the 45 studies provided a prospective model-guided impact evaluation demonstrating that implementation changed clinical management, patient outcomes, or resource use. Accordingly, model maturity was interpreted using validation setting, calibration, discrimination, threshold-dependent performance, and decision-analytic evidence rather than AUROC alone.

### 3.3. Model-Development Quality, Risk of Bias, and Applicability

PROBAST + AI assessments distinguished methodological quality of model development from risk of bias in model evaluation. Forty-four of the 45 included studies contained an eligible model-development component; El Hechi et al. [21] represented validation of an existing model and was therefore assessed only for model evaluation. Among the 44 model-development assessments, 10 (22.7%) were judged low concern, 24 (54.5%) unclear, and 10 (22.7%) high overall.

For the principal model evaluation in each of the 45 included studies, overall risk of bias was judged low in 8 studies (17.8%), unclear in 27 (60.0%), and high in 10 (22.2%). At the domain level, participants and data sources were judged low risk in 43/45 studies (95.6%) and high risk in 2/45 (4.4%). Predictors were low risk in 44/45 (97.8%) and high risk in 1/45 (2.2%). The outcome domain was low risk in 36/45 studies (80.0%), unclear in 8/45 (17.8%), and high risk in 1/45 (2.2%). The analysis domain accounted for the largest proportion of concerns, with 9/45 studies (20.0%) judged low risk, 27/45 (60.0%) unclear, and 9/45 (20.0%) high risk.

Applicability was assessed separately from methodological risk of bias. For the principal model evaluations, applicability to the review question was judged low concern overall in 30/45 studies (66.7%) and high concern in 15/45 (33.3%). By applicability domain, participants and data sources were judged low concern in 40/45 studies (88.9%) and high concern in 5/45 (11.1%); predictors were low concern in 41/45 (91.1%) and high concern in 4/45 (8.9%); and outcomes were low concern in 37/45 (82.2%) and high concern in 8/45 (17.8%). Applicability concerns primarily reflected mixed surgical populations without separately extractable abdominal-surgery performance, predictor timing that did not fully correspond to the intended clinical use, and outcomes or prediction tasks that were indirect relative to the principal review question.

**Figure 2:**
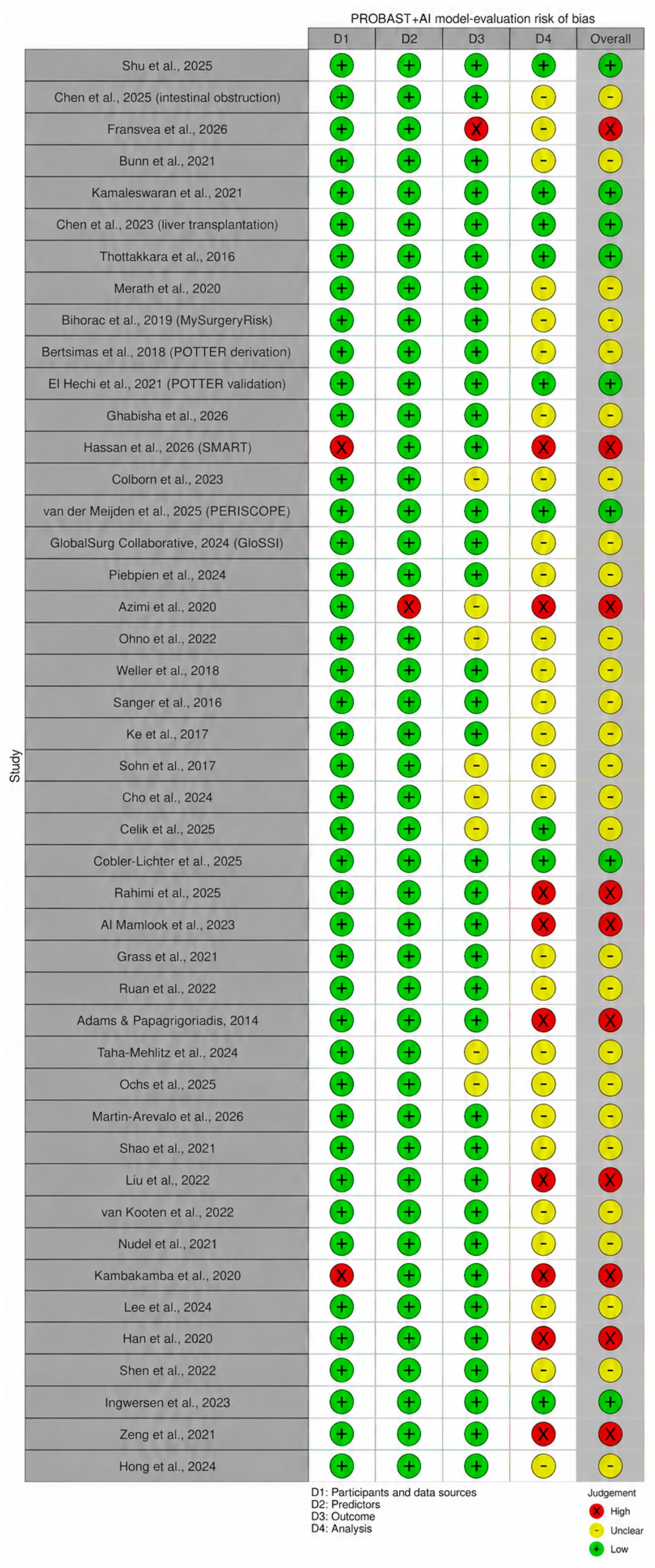
PROBAST + AI model-evaluation risk-of-bias assessment across the 45 included studies [1,2,3,4,5,6,7,8,9,10,19,20,21,22,23,24,25,26,27,28,29,30,31,32,33,34,35,36,37,38,39,40,41,42,43,44,45,46,47,48,49,50,51,52,53]. Risk of bias was assessed across four domains: D1, participants and data sources; D2, predictors; D3, outcome; and D4, analysis. Green symbols indicate low risk of bias, yellow symbols indicate unclear risk, and red symbols indicate high risk. The overall evaluation judgment was classified as high when at least one domain was judged high, unclear when no domain was high but at least one was unclear, and low when all four domains were judged low. Detailed signaling-question responses, supporting source locations, model-development quality assessments, and applicability judgments are provided in Supplementary Table S4 [1,2,3,4,5,6,7,8,9,10,19,20,21,22,23,24,25,26,27,28,29,30,31,32,33,34,35,36,37,38,39,40,41,42,43,44,45,46,47,48,49,50,51,52,53].

High overall evaluation risk of bias was assigned only when a documented methodological limitation provided a plausible mechanism for biased performance estimation; small sample size, retrospective design, or absence of geographic external validation alone was not considered sufficient for a high-risk judgment. Detailed signaling-question responses, supporting source locations, model-development quality judgments, model-evaluation risk-of-bias assessments, and applicability assessments are provided in Supplementary Table S4. Figure 2 presents the study-level PROBAST + AI model-evaluation risk-of-bias assessment for the principal evaluation in each of the 45 included studies.

### 3.4. Predictive Performance by Clinical Outcome

#### 3.4.1. Postoperative Sepsis and Septic Shock

Eleven studies evaluated sepsis-related outcomes: six evaluated a sepsis-related endpoint as the primary modeled outcome [1,2,3,20,26,27], whereas five incorporated sepsis or septic shock within broader postoperative-complication frameworks [21,24,25,28,29]. Outcome definitions among the six primary-target studies were materially heterogeneous. Three studies consistently used Sepsis-3-based endpoints [2,26,27]. Shu et al. used an ICD-10-coded composite of sepsis, severe sepsis, or septic shock in the development/internal-validation cohorts, whereas the MIMIC-IV external-validation cohort used postoperative Sepsis-3 within 30 days [1]. Bunn et al. used an ACS-NSQIP definition of postoperative sepsis or septic shock based on documented infection or end-organ ischemia together with systemic inflammatory-response criteria [20]. Fransvea et al. modeled qSOFA ≥ 2 versus <2; although the authors described the target as postoperative sepsis and referenced Sepsis-3, this endpoint was treated in the present review as a qSOFA-based sepsis-risk proxy rather than an independently established consensus-defined sepsis endpoint [3].

Shu et al.’s abdominal-surgery KNN/SVM ensemble achieved an AUROC of 0.892 in internal testing and 0.782 in external validation [1]; interpretation of this change also requires recognition that outcome ascertainment differed between the development and external-validation cohorts. Chen et al.’s intestinal-obstruction SVC model predicted Sepsis-3 within 7 postoperative days and achieved an internal AUROC of 0.876 (sensitivity 0.734). AUROCs in the three individual external cohorts were 0.873, 0.896, and 0.926, while the merged external evaluation yielded an AUROC of 0.863 (95% CI 0.832–0.892) [2]. Fransvea et al. enrolled 2571 patients, of whom 2563 were analyzed and 119 had qSOFA ≥ 2. The ensemble achieved an AUROC of 0.961 (95% CI 0.937–0.986), sensitivity of 0.774, specificity of 0.965, and accuracy of 0.956 [3]. The primary report also contained a conflicting secondary description of sensitivity and specificity; because those values were inconsistent with the reported outcome prevalence and confusion-matrix results, the internally coherent nested-cross-validation estimates reported in the Results were used.

In 223,214 appendectomies, Bunn et al. evaluated ACS-NSQIP-defined postoperative sepsis/septic shock; LR, RF, and XGBoost achieved AUROCs of approximately 0.70, SVM 0.51, and the combined ensemble 0.71 (95% CI 0.69–0.73) [20]. Kamaleswaran et al. serially identified Sepsis-3 onset and used continuous physiological data for dynamic early warning; at 12 h before meeting Sepsis-3 criteria, the XGBoost model achieved an AUROC of approximately 0.97 with sensitivity 0.94 and specificity 0.90, although evaluation was limited to internal cross-validation [26]. Chen et al. predicted Sepsis-3 within 7 days after liver transplantation using pre- and intraoperative information; the selected random forest model achieved an AUROC of 0.731 in internal validation and 0.755 in a temporally independent validation cohort [27].

Broader postoperative-complication frameworks provided additional contextual evidence. Thottakkara et al. (*n* = 50,318) reported discrimination ranging from 0.757 to 0.909 across algorithms for severe sepsis [24], and Merath et al. (*n* = 15,657) reported a c-statistic of 0.79 for sepsis within a broader complication model [28]. MySurgeryRisk was evaluated in 51,457 patients across eight postoperative complications, including sepsis; the reported overall discrimination range of 0.82–0.94 represented performance across the eight complications and was therefore not interpreted as a sepsis-specific performance range [25]. POTTER was derived in 382,960 emergency-surgery cases [29] and independently validated in 59,955 emergency general-surgery patients, with reported c-statistics of 0.93 for septic shock overall and 0.87 in the emergency-laparotomy subgroup [21].

Overall, discrimination ranged from modest to high, but direct numerical comparison was limited by differences in population, prediction time, validation design, and, importantly, outcome ascertainment. The six primary sepsis-related studies did not evaluate an identical clinical endpoint: their targets included consensus-defined Sepsis-3, administrative/coded or older SIRS-based definitions, and a qSOFA-based proxy. Performance estimates were therefore interpreted according to the specific outcome definition and evaluation setting rather than as interchangeable estimates of postoperative sepsis prediction. External performance was also variable, with Shu et al. declining from an AUROC of 0.892 to 0.782 while the intestinal-obstruction model remained above 0.87 across external cohorts [1,2]. These differences precluded ranking models by discrimination alone.

#### 3.4.2. Postoperative Infection and Surgical-Site Infection

Nineteen studies addressed postoperative infection/SSI after abdominal or general surgery [4,5,8,9,19,22,30,31,32,33,34,35,36,37,38,39,40,41,42], with targets ranging from composite infection or any SSI to superficial, deep, organ-space, and surveillance-detected infection. Ghabisha et al. (*n* = 525) reported AUROC 0.934 for XGBoost and 0.924 for random forest, but calibration showed risk overestimation despite favorable decision-curve findings [4]. SMART (*n* = 2716) achieved AUROC 0.86 and sensitivity 96.9% with internal evaluation only [8].

Colborn et al. analyzed 30,639 operations across five hospitals; models exceeded AUROC 0.91 with sensitivities > 87% and specificities >81% in a chronologically held-out cohort [30]. PERISCOPE (*n* = 253,010) achieved external AUROCs around 0.77 and 0.85; local updating followed by temporal validation improved discrimination to about 0.82 and 0.91, with better calibration and decision-analytic performance [9]. GloSSI was developed in 14,019 and externally validated in 8464 gastrointestinal-surgery patients, with AUROCs 0.738 and 0.730 and external calibration intercept −0.098/slope 1.008 [5]. Piebpien et al. reported AUROC 0.768 for an updated SENIC logistic model versus 0.679 for naive Bayes, illustrating that ML was not uniformly superior [31].

Dynamic postoperative prediction and early-detection models used accumulating clinical information. Sanger et al. improved SSI discrimination from approximately 0.67 at baseline to 0.76 with serial wound and physiological data (sensitivity 0.80; NPV 0.93) [19].

Contextual evidence from retrospective surveillance and case ascertainment. Three studies primarily evaluated AI/ML-assisted postoperative SSI surveillance or case ascertainment rather than conventional prediction of a future complication [36,37,38]. Sohn et al. used a Bayesian-network approach to detect clinically important colorectal SSI, achieving an AUROC of 0.827 compared with 0.719 for logistic regression [36]. Cho et al. reported an AUROC of 0.963 for colon-SSI surveillance; at 95% sensitivity, PPV was 21.1%, increasing to 28.9% with rule-based screening, while the estimated manual chart-review workload was reduced by 83.9% [37]. Celik et al. similarly evaluated postoperative colon-SSI surveillance using structured EHR data together with NLP-derived clinical-note features [38]. These studies were retained as contextual evidence relevant to automated surveillance and case finding; their performance was not interpreted as evidence of clinically actionable pre-event prediction unless prediction before outcome onset or a clinically meaningful lead time was demonstrated.

Procedure-specific analyses included trauma laparotomy, where Cobler-Lichter et al. reported AUROCs 0.805 overall, 0.832 for organ-space SSI, and 0.776 for deep SSI [22], and a 2,882,526-operation cohort in which Al Mamlook et al. reported AUROC 0.8518 for a deep neural network under 10-fold cross-validation [40]. Ruan et al.’s GRU-D longitudinal model showed that performance and predictor contribution changed with infection type and postoperative time [42].

Overall, infection/SSI studies addressed clinically distinct tasks ranging from perioperative risk prediction to dynamic postoperative prediction or early detection and retrospective surveillance/case ascertainment. Reported discrimination therefore could not be interpreted without considering predictor timing, outcome definition, intended use, and validation strategy. Surveillance performance and reductions in manual chart-review burden were treated as operational case-finding outcomes rather than evidence of pre-event clinical warning. Among predictive models, interpretation emphasized temporal ordering of predictors and outcomes, validation strategy, calibration, and clinically useful lead time rather than peak AUROC alone.

#### 3.4.3. Anastomotic Leakage

Eight studies predicted anastomotic/gastrointestinal leakage after colorectal, gastric, esophagogastric, or bariatric surgery, using preoperative, perioperative, or early postoperative information [6,10,23,43,44,45,46,47]. Adams and Papagrigoriadis’ small colorectal neural-network study (*n* = 76) achieved internal AUC 0.89, sensitivity 85.0%, specificity 82.1%, with 83.3% specificity in a 12-patient prospective pilot [43]. In four Swiss centers, Taha-Mehlitz et al. reported random forest AUC 0.78 internally but 0.60 externally; logistic regression was more stable at 0.69 and 0.61 [44].

Ochs et al. analyzed 9120 colorectal resections using a cross-attention meta-model, which achieved an AUC of 0.89 (95% CI 0.83–0.95) in cross-validation and 0.78 in external validation [6]. Martin-Arevalo et al.’s 24 h postoperative inflammatory model achieved an AUC of 0.819 in development and was subsequently evaluated in a prospective temporal-validation cohort of 250 patients, in which seven anastomotic leaks occurred (2.8%; non-event prevalence 97.2%) [10]. At the prespecified probability threshold of 0.1258, the model achieved an AUROC of 0.73 (95% CI 0.54–0.92), sensitivity of 57.1%, specificity of 60.1%, positive predictive value of 4.0%, and negative predictive value of 97.9%, with three of the seven leaks missed. The reported calibration slope was 0.987, the calibration intercept was approximately 0.505, and the Brier score was approximately 0.094; decision-curve analysis showed positive net benefit across thresholds of approximately 5–20%. The high negative predictive value should be interpreted against the baseline non-event prevalence of 97.2% and therefore does not alone establish strong rule-out performance. Although the calibration slope was close to one, the positive calibration intercept indicates systematic underestimation of risk; calibration should therefore not be described as near ideal.

After gastrectomy, Shao et al. reported random forest AUC 0.89 with sensitivity 81.8% and specificity 82.2% and implemented a web tool [45]; Liu et al. reported SVM AUROC 0.89 versus 0.86 for postoperative-day-4 CRP alone [46]. In 4228 esophageal and 2199 gastric resections, van Kooten et al. found no significant ML advantage: generalized linear regression achieved AUC 0.619 for esophageal leakage and 0.680 after gastrectomy, comparable with neural networks/LASSO/SuperLearner [47]. In 436,807 bariatric procedures, Nudel et al. reported leak AUCs 0.75 for ANN, 0.70 for XGBoost, and 0.63 for logistic regression [23].

Overall, several internally evaluated models approached AUC 0.90 [43,45,46], but external performance could fall substantially [6,44]. The temporally validated 24 h model retained moderate discrimination, but its high negative predictive value occurred in a cohort with a 97.2% non-event prevalence, while the positive calibration intercept indicated systematic risk underestimation despite a calibration slope close to one [10]; conventional regression matched or exceeded ML in the large esophagogastric cohort [47]. Pre/perioperative risk prediction and early postoperative detection were therefore interpreted as distinct tasks.

#### 3.4.4. Postoperative Pancreatic Fistula

Five studies evaluated POPF after pancreaticoduodenectomy using preoperative CT/clinical data, perioperative variables, or serial postoperative measurements [7,48,49,50,51]. Kambakamba et al.’s proof-of-principle case–control study (55 POPF/55 controls) reported CT-texture AUC 0.95, sensitivity 96%, and specificity 98% versus risk-score AUCs 0.76 and 0.72, but only 10-fold cross-validation [48]. Lee et al. (*n* = 1333) combined clinical/body-composition data with CT deep learning; in a temporal holdout, the ensemble achieved AUC 0.75 (95% CI 0.71–0.80) for any POPF and 0.68 (0.58–0.78) for clinically relevant POPF [7].

Han et al. (*n* = 1769; 221 events) reported maximum AUC 0.74 for a neural network with recursive feature elimination and implemented a web platform, but without independent validation [49]. Shen et al. (*n* = 2421; 424 grade B/C events) used postoperative information; CatBoost achieved mean AUC 0.81 (95% CI 0.80–0.82) in repeated cross-validation and about 0.83 in random test sets, with early drain-fluid amylase the dominant feature [50].

In the largest cohort, Ingwersen et al. analyzed 4912 patients from 16 Dutch centers (799 clinically relevant POPF) and found near-equivalent bootstrap-validated performance: gradient boosting AUROC 0.74 (IQR 0.73–0.74), Brier 0.13, versus logistic regression AUROC 0.73, Brier 0.12 [51].

Accordingly, the highest discrimination arose from a small internally validated imaging study, whereas larger models generally achieved AUCs around 0.68–0.83 [7,48,49,50]. Postoperative information improved early-diagnostic performance [50], and complex ML did not clearly outperform logistic regression in the largest multicenter cohort [51]. Pre/perioperative risk prediction and postoperative early diagnosis were therefore considered separately.

#### 3.4.5. Other Major Abdominal Postoperative Complications

Two studies modeled broader composite postoperative complications and were analyzed separately because their endpoints combined infectious and noninfectious events [52,53]. Zeng et al. studied 175 liver-resection patients (58 complications): a C5.0 tree achieved AUC 0.91 (95% CI 0.77–1.00), accuracy 92.45%, sensitivity 87.5%, and specificity 94.59%, while random forest had the most favorable decision-curve profile; operative duration, BMI, and incision length were prominent predictors [52].

Hong et al. developed models for serious 30-day complications after laparoscopic radical gastrectomy in 998 patients across 16 centers and externally validated them in 398 patients. The composite included leakage, pulmonary/abdominal infection, wound complications, obstruction, bleeding, pancreatic fistula, sepsis, and other events [53]. Random forest performed best overall: AUC 0.897 development/0.752 external for total gastrectomy and 0.885/0.903 for distal gastrectomy [53].

Thus, broad-complication discrimination ranged from moderate to high, but the liver-resection estimate came from a small internally split cohort whereas gastrectomy underwent independent external validation [52,53]. Because these were heterogeneous composites, they were kept separate from outcome-specific sepsis, infection, leakage, and POPF models.

### 3.5. Validation, Calibration, and Clinical Utility

Validation approaches varied substantially across studies. Fifteen of 45 studies (33.3%) provided a strict temporal, geographic/external-center, or independent-investigator evaluation of the principal model, whereas the remaining studies relied predominantly on internal splitting, cross-validation, bootstrap resampling, or other non-independent evaluation strategies. External performance was not uniformly preserved. Shu et al. declined from an AUROC of 0.892 in internal testing to 0.782 in external validation [1], whereas the intestinal-obstruction model showed external AUROCs ranging from 0.863 to 0.926 across individual and merged external evaluations [2]. The liver-transplant random forest showed similar discrimination in internal and temporal validation (0.731 vs. 0.755) [27], and GloSSI remained relatively stable between derivation and external validation (0.738 vs. 0.730) [5]. In contrast, the Taha-Mehlitz random forest declined from 0.78 internally to 0.60 externally [44], and the Martin-Arevalo model declined from 0.819 in development to 0.73 (95% CI 0.54–0.92) in temporal validation [10].

Explicit calibration analysis was reported in 9 of 45 studies (20.0%), while 12 of 45 (26.7%) provided any calibration-related information. Reported calibration measures included calibration plots, calibration intercepts or calibration-in-the-large, slopes, and observed-to-expected comparisons. For example, GloSSI showed an external calibration intercept of −0.098 and slope of 1.008 [5], whereas Martin-Arevalo reported a slope of 0.987 with an intercept of approximately 0.505 [10]. Ghabisha et al. demonstrated appreciable calibration departures despite high discrimination [4]. Brier scores were considered separately as measures of overall probabilistic prediction error rather than calibration alone; for example, the intestinal-obstruction model reported a Brier score of 0.108 [2], while the temporally validated Martin-Arevalo model reported a Brier score of approximately 0.094 [10].

Decision-curve analysis or another net-benefit assessment was reported in 7 of 45 studies (15.6%). These analyses provided information beyond discrimination by evaluating whether model-based decisions could offer net benefit across clinically relevant probability thresholds. However, no study (0/45) provided a prospective model-guided impact evaluation demonstrating that use of the model changed clinical management, patient outcomes, or resource utilization. Some studies reported operational or implementation-oriented features, including reduction in manual review burden [37] and web-based calculators [45,49], but such tools were not considered evidence of prospective clinical impact. Overall, model maturity was therefore interpreted using independent or temporal validation, calibration, probabilistic performance, threshold-dependent performance, and decision-analytic evidence rather than AUROC alone.

### 3.6. Predictors and Feature Importance

Predictors spanned demographics/comorbidity, laboratory and inflammatory markers, physiological status, operative characteristics, longitudinal postoperative observations, EHR variables, and imaging features. Selection/interpretation methods included regularization, recursive feature elimination, algorithm-derived importance, and SHAP; because definitions, timing, and importance measures were not standardized, predictor effects were synthesized qualitatively by clinical domain and perioperative stage.

For sepsis, recurrent features reflected baseline reserve, inflammation, and perioperative physiological disturbance: albumin, ASA class, neutrophil-to-lymphocyte ratio, age, glucose [1]; age, ASA, severity measures, shock index, and fluid variables [2]; transfusion, blood loss, anesthesia duration, bilirubin, fluids, and urine output after liver transplantation [27]; and dynamic respiratory rate, systolic pressure, and heart-rate changes before Sepsis-3 criteria [26]. Infection/SSI models similarly emphasized operative complexity, comorbidity, ASA status, emergency surgery, obesity, and physiological burden [4,40], with trauma-specific injury/transfusion features [22] and colorectal hematologic, inflammatory, renal, and operative variables [32,39].

Postoperative surveillance increasingly relied on evolving information: serial wound and physiological observations [19], NLP-derived SSI terms plus structured recovery variables [38], and longitudinal measurements approaching infection onset [35,42]. Early leak models emphasized platelet-to-lymphocyte ratio, monocytes, systemic inflammatory indices, CRP, operative duration, and approach [10], or CRP, albumin, NLR, and immunonutritional indices after gastric surgery [46], whereas purely preoperative leak models used baseline patient/procedural factors [6,44].

POPF prediction was more anatomical and procedure specific, repeatedly using duct diameter, gland texture, BMI, albumin, operative characteristics, and CT-derived pancreatic/body-composition features [7,48,49]; with postoperative data, drain-fluid amylase became dominant while duct size, tumor size, and texture remained relevant [50]. Across outcomes, preoperative models emphasized baseline reserve and inflammation, perioperative models procedural burden and physiology, and postoperative models dynamic inflammatory, wound, physiological, or drain signals [1,19,27,50]. No single predictor set dominated across all populations or outcomes.

### 3.7. Quantitative Synthesis and Heterogeneity

Clinical and methodological heterogeneity remained substantial in population, outcome and definition, prediction horizon/timing, data modality, model development, validation stage, and performance metric. Primary sepsis-related studies differed not only in prediction timing but also in endpoint ascertainment, ranging from Sepsis-3-defined postoperative events to ICD-coded or SIRS-based sepsis/septic-shock definitions and a qSOFA-based proxy; prediction tasks ranged from preoperative risk estimation to dynamic forecasting shortly before Sepsis-3 onset [1,2,3,20,26,27]; infection studies included composite infection and multiple SSI definitions from baseline prediction to longitudinal surveillance [4,19,22,30]; leakage spanned colorectal, gastric, esophagogastric, and bariatric procedures [10,23,44,47]; and POPF varied by target and whether predictors were imaging, perioperative, or postoperative [7,48,49,50]. Reporting also mixed AUROC, classification metrics, AUPRC, F1, Brier/calibration, and net benefit across development, internal, temporal, and external validation stages [5,6,9,53], often with correlated algorithms from the same cohort.

No subgroup therefore met the prespecified comparability criteria for quantitative pooling. Pooled AUROC, sensitivity, specificity, or summary ROC estimates were not calculated because combining internal with external validation or preoperative risk models with postoperative early-warning models would merge clinically distinct tasks. Results were synthesized narratively by outcome, prediction timing, data modality, and validation strategy, emphasizing preservation of performance in temporal/external validation and the presence of calibration or decision-analytic assessment [1,5,9,10].

#### Narrative Sensitivity Analyses

Narrative sensitivity analyses were undertaken to examine whether the principal interpretation changed under progressively more restrictive evidence definitions. Restriction to studies reporting abdominal/general-surgery-specific performance retained 38 of 45 studies, and the overall interpretation was unchanged. Restriction to genuine pre-event prognostic prediction or postoperative early-warning evaluations likewise retained 38 of 45 studies and did not materially alter the findings.

Within the six primary sepsis-related studies, exclusion of the qSOFA-based proxy study retained five of six studies and did not change the overall interpretation that predictive performance was promising but heterogeneous and incompletely validated. Restriction further to evidence based on strict Sepsis-3 outcome definitions yielded four studies or evaluation cohorts [1,2,26,27]; this substantially narrowed the evidence base but did not alter the cautious conclusion regarding clinical readiness.

When the synthesis was restricted to models undergoing strict temporal, geographic/external-center, or independent-investigator evaluation, 15 of 45 studies remained. Transportability continued to vary, with some models maintaining discrimination and others showing appreciable performance deterioration outside their development setting. Restriction to the eight studies judged at low overall model-evaluation risk of bias using PROBAST + AI resulted in a substantially smaller evidence base but supported the same cautious interpretation. Conversely, exclusion of the 10 studies judged at high evaluation risk of bias retained 35 of 45 studies and did not materially alter the overall conclusions. Detailed study-level inclusion in each sensitivity analysis and linked-cohort considerations are provided in Supplementary Table S5.

### 3.8. Reporting Bias and Certainty of Evidence

Because no outcome domain contained at least 10 sufficiently comparable studies for quantitative synthesis, funnel plots and regression-based tests for small-study effects were not performed. Publication or reporting bias was therefore considered not formally assessable for all four graded outcome domains and was not assumed to be absent.

Using the protocol-specified GRADE principles operationalized for prediction-model evidence as described in Section 2.9, certainty in predictive performance and transportability was judged very low for primary postoperative sepsis/septic-shock prediction, low for postoperative infection/surgical-site infection, very low for anastomotic leakage, and very low for postoperative pancreatic fistula.

For primary sepsis/septic-shock prediction, risk of bias was judged serious because the six principal studies included three evaluations at low, two at unclear, and one at high overall PROBAST + AI evaluation risk of bias. Inconsistency was serious because outcome definitions, prediction timing, clinical tasks, validation strategies, and reported performance varied substantially. Indirectness was also serious: the evidence included consensus Sepsis-3 outcomes alongside ICD-coded or older SIRS/ACS-NSQIP definitions and a qSOFA-based proxy, and restriction to strict Sepsis-3 evidence reduced the evidence base to four studies or evaluations. Imprecision was serious because several clinically important validation estimates were based on relatively few events or had wide confidence intervals, despite some larger development datasets.

For postoperative infection/SSI, risk of bias was serious because only two of 19 principal evaluations were judged at low overall PROBAST + AI evaluation risk of bias, while 13 were unclear and four were high. Inconsistency was serious because studies differed substantially in infection definition, prediction horizon, surveillance versus prognostic use, surgical population, validation design, and observed discrimination. Indirectness was not downgraded at the body-of-evidence level because most studies directly evaluated postoperative infection or SSI in abdominal or general surgical populations, and restriction to abdominal-specific and genuine pre-event evidence did not materially alter the interpretation. Imprecision was not considered serious overall because several large temporal or external validation cohorts provided relatively precise performance estimates, although individual smaller studies remained imprecise.

For anastomotic leakage, risk of bias was serious because none of the eight principal evaluations was judged at low overall evaluation risk of bias; six were unclear and two were high. Inconsistency was serious because models addressed different operations, prediction time points, predictor sets, and validation settings, with materially different discrimination across internal and external evaluation. Indirectness was not considered serious overall because the studies directly evaluated anastomotic or gastrointestinal leakage after abdominal surgery. Imprecision was serious because important external or temporal validations contained few leakage events or wide uncertainty; notably, the prospective temporal validation of the Martin-Arevalo model included seven leaks and yielded an AUROC of 0.73 with a 95% CI of 0.54–0.92.

For postoperative pancreatic fistula, risk of bias was serious because two of five principal evaluations were high risk, two were unclear, and only one was low risk. Inconsistency was serious because studies differed in the definition of any versus clinically relevant fistula, predictor timing, imaging versus clinical inputs, and model performance. Indirectness was not considered serious overall because all five studies directly addressed pancreatic fistula after pancreatic surgery. Imprecision was serious because independently validated evidence remained sparse, some relevant evaluation cohorts contained relatively few outcome events, and uncertainty around clinically relevant fistula prediction remained substantial.

Five broader postoperative-complication studies in which sepsis was one component of a multisystem or composite prediction framework [21,24,25,28,29] and two additional broad postoperative-complication models [52,53] were retained as contextual evidence but were not assigned separate GRADE certainty ratings. Their reported performance could not be mapped cleanly to one of the four principal outcome-specific claims without combining clinically heterogeneous endpoints. Validation setting and applicability informed the conventional GRADE domains where relevant but were not treated as additional independent downgrading domains. Detailed domain-level judgments are presented in Table 2.

## 4. Discussion

### 4.1. Principal Findings

This review of 45 studies found that postoperative AI/ML performance depended at least as much on the clinical prediction task, timing, population, and validation setting as on algorithm choice. Primary sepsis-related models ranged from preoperative risk estimates to dynamic forecasts shortly before onset, and external performance was not uniform: Shu et al. attenuated on independent validation whereas Chen et al.’s intestinal-obstruction model remained comparatively stable [1,2], with liver-transplant studies addressing different prediction windows [26,27].

The same pattern appeared in infection and surgically specific complications. GloSSI retained similar derivation/external discrimination, while PERISCOPE showed cross-hospital variation and benefit from local updating [5,9]; some colorectal leak models deteriorated externally, whereas temporal validation of the early postoperative inflammatory model demonstrated moderate discrimination with a high negative predictive value that required interpretation in the context of the very low event prevalence [10]. These findings reinforce that a high AUROC must be interpreted in relation to where, when, and in whom it was estimated. The principal interpretation remained unchanged in narrative sensitivity analyses restricted to abdominal-specific populations, genuine pre-event prediction or early-warning evaluations, models undergoing temporal, geographic/external-center, or independent evaluation, and studies remaining after exclusion of evaluations judged at high risk of bias.

Greater algorithmic complexity also did not consistently improve clinically relevant performance. Gradient boosting and logistic regression were nearly equivalent for clinically relevant POPF [51], conventional regression matched or exceeded several ML approaches in esophagogastric surgery [47], and complex SSI models did not always retain their development advantage externally [5]. Model value therefore reflects discrimination, calibration, generalizability, predictor availability, interpretability, and the consequences of acting on a prediction rather than an ‘AI versus statistics’ distinction.

Finally, the evidence formed a temporal continuum: preoperative models estimate baseline susceptibility, perioperative models add operative burden and physiological disturbance, and dynamic postoperative models incorporate evolving physiological, inflammatory, wound, or drain information [10,19,26,50]. Later models may discriminate better because they observe information closer to the event. The most mature studies extended beyond internal discrimination to temporal/external validation, calibration, and net-benefit assessment [1,5,9,10], but the available evidence remained insufficient to support routine AI/ML model use across major abdominal surgical populations.

### 4.2. Prediction Timing and the Clinical Role of AI

Clinical value depends on when a prediction is available relative to the complication. Preoperative, perioperative, and dynamic postoperative models answer different questions, use different information, and provide different windows for action. Preoperative models use demographics, comorbidities, laboratories, nutritional/inflammatory status, and planned procedure characteristics to support counseling, optimization, prevention, and surveillance planning [1,4,7,44], but cannot capture operative complexity, blood loss, transfusion, instability, or the early response to surgery.

Perioperative models add operative duration, blood loss, transfusion, fluid balance, procedural complexity, and intraoperative physiology [2,27,45,51], potentially informing immediate postoperative disposition or monitoring. Dynamic models move closer to the event: physiological trajectories forecast liver-transplant sepsis [26], serial wound/physiological data improved SSI detection [19,42], inflammatory markers informed early leak prediction [10], and drain-fluid amylase became dominant for clinically relevant POPF [50].

This creates an accuracy-lead-time trade-off. Later models may discriminate better because early manifestations are already emerging, but leave less time for intervention; earlier models may be less accurate yet offer more opportunity for prevention, intensified surveillance, diagnostic work-up, or escalation. This distinction is particularly important for SSI, leakage, and fistula: they are not sepsis, but earlier recognition may identify pathways preceding intra-abdominal infection and systemic deterioration.

Future studies should therefore prespecify prediction time, horizon, intended action, and minimum clinically useful lead time, and report performance at clinically meaningful time points. A sequential strategy that updates risk from preoperative stratification through perioperative reassessment to dynamic postoperative warning [9,26,42,50] may better reflect evolving postoperative risk than a single static estimate.

### 4.3. Validation, Calibration, and Translation to Clinical Utility

A major translational gap was the reliance on internal validation. Cross-validation, bootstrap resampling, and random splits estimate performance within the available data but do not establish transportability. Several models changed materially in independent or temporal populations [1,10,44,53], whereas others retained broadly similar performance across external or temporal cohorts, including the intestinal-obstruction model, the liver-transplant random forest, and GloSSI [2,5,27]. Transportability must therefore be demonstrated rather than inferred.

PERISCOPE further showed that external validation may lead to local updating: performance differed across hospitals and improved after adaptation to local historical data [9]. Because case mix, coding, laboratory workflows, procedure mix, and prevalence differ by institution, recalibration or updating may be necessary, but any adapted model requires renewed validation.

Calibration is distinct from discrimination and was less consistently reported. GloSSI showed close external calibration, whereas highly discriminative gastrointestinal SSI models demonstrated appreciable calibration departures [4,5]. The temporally validated colorectal-leak model reported a calibration slope close to one, but its positive calibration intercept indicated systematic underestimation of risk, illustrating why slope and intercept should be interpreted jointly rather than describing calibration as near ideal [10]. Thus, good ranking does not guarantee probabilities suitable for threshold-based decisions. Decision-curve analyses in selected studies estimated potential net benefit [1,4,9,10], but analytical utility is not evidence that implementation improves outcomes; likewise, a web calculator demonstrates accessibility, not impact [45,49].

Operational evidence was limited but informative: ML-assisted SSI surveillance improved PPV and reduced estimated chart-review workload [37]. Clinical translation should therefore progress from internal validation to temporal/external validation, calibration, decision-threshold and workflow assessment, and ultimately prospective impact studies testing whether model-guided care changes management, outcomes, or resource use.

### 4.4. Predictor Patterns, Interpretability, and Model Complexity

Predictive information was multidimensional, spanning physiological reserve, comorbidity, inflammatory/nutritional status, operative burden, and evolving postoperative physiology. Primary sepsis-related models repeatedly selected albumin, ASA status, inflammatory indices, age, glucose, transfusion/blood loss, fluid balance, and vital-sign trajectories [1,26,27]. Predictor importance shifted with timing: preoperative models emphasized baseline patient/procedure data [1,32,33,44], perioperative models operative burden and physiology [2,27,45,51], and postoperative models serial physiological, wound, inflammatory, and drain-derived data [10,19,42,50].

Procedure-specific anatomy added further signal in POPF, including duct diameter, gland characteristics, body composition, and CT-derived features [7,48,49]. Such imaging and multimodal inputs may add information but also require standardized acquisition, preprocessing, and validation across scanners and institutions before generalization can be assumed.

Feature-importance methods—including random forest/boosting importance, recursive feature elimination, and SHAP—describe contributions within a specific model and dataset, not causality. Rankings can shift with predictor correlation, missingness, measurement frequency, feature engineering, and institutional practice, as reflected by variation across SSI populations [4,22,39,40]. Reduced predictor sets, feature rankings, and web tools may aid use [38,45,49], but clinically meaningful interpretability also requires knowing when predictors are available, whether probabilities are calibrated, and what action follows a given risk.

More complexity was not inherently better: generalized linear regression matched or exceeded several ML methods in esophagogastric surgery [47], and gradient boosting and logistic regression were almost equivalent for POPF [51]. Although flexible algorithms can capture nonlinearities and interactions that simpler models may miss, added complexity should be justified by demonstrable incremental performance during appropriate validation. Future models should therefore prioritize clinically coherent, reproducibly measured predictors available at the intended decision point and justify dynamic, imaging, NLP, or multimodal inputs through reproducible incremental value over simpler baselines.

### 4.5. Methodological Limitations of the Current Evidence Base

The evidence base remained methodologically heterogeneous. Many studies were retrospective, single-center, registry based, or derived from routine EHRs, with fewer rigorous temporal or independent external validations [1,4,9,49]. Such datasets enable scale but can encode institution-specific selection, workflows, coding, predictor availability, and outcome ascertainment, so development performance cannot be assumed to generalize.

Sample size alone did not guarantee reliability. Cohorts ranged from small procedure-specific studies to hundreds of thousands or millions of operations [20,23,40], yet rare outcomes could still yield few events; conversely, very high discrimination sometimes came from small cohorts [43,48]. Credibility therefore depends on event counts relative to model complexity, feature-selection stability, and validation rigor.

Outcome ascertainment was heterogeneous even within the primary sepsis-related evidence. Three studies consistently used Sepsis-3 [2,26,27], Shu et al. used ICD-10-coded sepsis/severe sepsis/septic shock during development but Sepsis-3 in external validation [1], Bunn et al. used an older ACS-NSQIP/SIRS-based definition [20], and Fransvea et al. modeled qSOFA ≥ 2 as a proxy endpoint [3]. These definitions should not be interpreted as interchangeable representations of postoperative sepsis. Outcome definitions and prediction targets also varied across composite infection, SSI subtypes, leakage, and fistula. Prediction timing added further heterogeneity because postoperative laboratory, documentation, wound, physiological, and drain data may represent increasingly proximal manifestations of the outcome [10,19,38,50]. Without explicit prediction times/horizons and predictor availability, high performance may reflect near-event detection rather than clinically useful early prediction.

Analytical pipelines differed in missing-data handling, feature selection, class imbalance, preprocessing, hyperparameter tuning, internal validation, and model selection; flexible algorithms and low-event datasets are particularly susceptible to optimism, and testing many algorithms can favor chance winners if development and evaluation are not separated. Reporting was also uneven: AUROC predominated while calibration, uncertainty, precision–recall, and threshold metrics were less consistent. For uncommon or imbalanced outcomes, good discrimination may coexist with low PPV, and overall accuracy may appear favorable despite inadequate sensitivity for the complication of interest [37,38]. These differences, reflected in PROBAST + AI, limited direct comparison and precluded defensible pooling; the main evidence gap is not absence of promising models but insufficiently standardized, independently validated evidence of transportability.

### 4.6. Clinical Implications and Future Research Priorities

The near-term role of perioperative AI is to support, not replace, clinical judgment through risk stratification, surveillance, and timely escalation at defined points in the surgical pathway. Intended use should be specified before development because preoperative optimization tools and postoperative surveillance systems require different predictors, timing, thresholds, and actions. Models should use information that is reproducibly available at the intended decision point and integrate with routine workflows; SMART, PERISCOPE, and SSI-surveillance studies illustrate potential pathways for perioperative assessment, monitoring/discharge support, and prioritization of manual review [8,9,37,38].

Before adoption, promising models require geographical and temporal external validation, calibration assessment, and transparent local updating when justified. PERISCOPE showed that adaptation could improve local discrimination, calibration, and net benefit [9], whereas GloSSI remained comparatively stable externally [5], indicating that the need for recalibration varies by model and setting.

Prospective evaluation should follow adequate retrospective/external validation. A silent deployment phase can test real-time data availability, technical reliability, workflow fit, alert burden, and live predictive performance before outputs influence care; SMART specifically proposed such an approach [8]. Subsequent impact studies should determine whether risk estimates alter clinician behavior, diagnostic timing, treatment, complications, resource use, or patient-centered outcomes.

Evaluation should also move beyond AUROC to calibration, threshold-specific sensitivity/specificity, predictive values, precision–recall for uncommon outcomes, and decision-analytic measures. Thresholds must reflect the consequences of false positives/negatives and the action triggered; a threshold suitable for chart-review prioritization may be inappropriate for invasive investigation, delayed discharge, or treatment escalation [9,37,38]. Clinical thresholds should be prespecified according to intended use rather than selected retrospectively to maximize reported performance.

Implementation is also a maintenance problem. Changes in populations, surgery, antimicrobials, laboratory workflows, and EHR systems can alter data distributions and prevalence; PERISCOPE explicitly considered performance/data drift [9]. Systems therefore need monitoring, criteria for recalibration/retraining, and validation of revised models. Equity should be assessed through prespecified subgroup discrimination and calibration because acceptable aggregate performance does not ensure comparable performance across patient groups; PERISCOPE’s subgroup analyses also highlighted missing race, ethnicity, and socioeconomic information [9].

Practical deployment also requires explicit local governance and technical oversight. Institutions should define clinical and technical ownership of the model, responsibilities for performance surveillance, criteria for recalibration or retraining, version control, audit trails, and circumstances requiring temporary suspension or retirement of a model. EHR integration should be evaluated for local variable mapping, measurement units, missingness, data latency, interface reliability, and alert routing, because changes in data pipelines or clinical workflows may alter both model inputs and downstream actions. Post-deployment monitoring should therefore include discrimination, calibration, event prevalence, data-quality and performance drift, alert burden, and clinically relevant subgroup performance. Material changes to the model, EHR configuration, or input-data pipeline should prompt documented reassessment and, where appropriate, revalidation before continued clinical use.

Future research should therefore shift from algorithm development toward clinically embedded evaluation with defined time points/horizons and responses, standardized outcomes, comparison with appropriate conventional baselines, multicenter/temporal validation, calibration, prospective workflow testing, and impact studies. Additional complexity from dynamic, imaging, NLP, or multimodal inputs should be justified by reproducible incremental value at a clinically useful time point.

### 4.7. Strengths and Limitations of This Review

This review’s main strengths were its clinically structured synthesis across primary sepsis-related outcomes, infection/SSI, leakage, POPF, and broader postoperative complications while keeping these outcomes distinct; its interpretation of models within preoperative, perioperative, and dynamic postoperative time points [10,19,26,50]; and its emphasis on validation, calibration, predictor availability, interpretability, and utility rather than headline AUROC alone. External performance was explicitly distinguished from development performance because transportability was preserved in some models but attenuated in others [1,5,9,44]. Avoiding quantitative pooling also prevented clinically distinct populations, outcomes, prediction horizons, and validation stages from being collapsed into misleading summary estimates.

The review also has limitations. Its broad eligibility framework introduced substantial clinical heterogeneity. Clinical sepsis ascertainment itself was heterogeneous, including consensus Sepsis-3 definitions, administrative/coded or older SIRS-based criteria, and a qSOFA-based proxy; consequently, the six primary sepsis-related studies were not treated as methodologically equivalent evidence of the same endpoint. SSI, leakage, and fistula are not surrogate definitions of sepsis; they provide contextual evidence but cannot establish primary sepsis-related prediction performance.

Searches covered PubMed, Scopus, and CENTRAL through 10 August 2026, so studies indexed only elsewhere or published later may have been missed. Because no subgroup was sufficiently comparable for pooling, summary accuracy estimates and conventional publication-bias tests were not produced.

Primary-study reporting was incomplete for calibration, prediction horizons, uncertainty, decision analysis, and threshold metrics, and outcome definitions/validation methods varied. Large registries or routine-care datasets may also overlap, so study sample sizes cannot be summed as unique patients. Finally, the literature predominantly evaluated model development or observational performance rather than prospective model-guided care; this review can therefore appraise predictive performance and methods but cannot establish that implementation improves diagnostic timing, management, outcomes, or resource use.

Accordingly, the review’s contribution is not a pooled estimate of AI performance but a structured appraisal of where perioperative prediction models stand, how their reported performance should be interpreted, and what evidence is still required before routine implementation.

## 5. Conclusions

Although selected AI/ML models demonstrated promising discrimination for postoperative sepsis and related complications, the available evidence remains insufficient to support routine AI/ML model use across major abdominal surgical populations. Across the 45 included studies, performance depended substantially on the clinical outcome, surgical population, prediction timing, predictor availability, and validation strategy; high development performance was not consistently maintained during temporal or external evaluation, and greater model complexity did not reliably outperform conventional approaches.

Postoperative infection, anastomotic leakage, and pancreatic fistula should remain distinct from primary sepsis-related prediction, while primary sepsis-related outcomes should be interpreted according to their underlying clinical definitions, ascertainment methods, and prediction tasks. Future models should therefore be anchored to a clearly defined clinical use case, prediction time and horizon, and intended clinical response, followed by rigorous temporal and multicenter external validation, assessment of calibration, and prospective model-guided impact studies. The most clinically valuable perioperative AI model will not necessarily be the one with the highest AUROC, but one that is reliable, calibrated, timely, transportable, interpretable, and demonstrably actionable in real-world surgical care.

## Figures and Tables

**Table 1:** Characteristics of the 45 included studies evaluating artificial intelligence and machine learning models for postoperative sepsis and related complications after major abdominal surgery.

Study [Ref]	Country/Setting	Study Design	Surgical Population; N	Modeled Outcome	Prediction Timing	AI/ML Approach	Validation Strategy
**Primary sepsis-related outcomes**							
Shu et al., 2025 [1]	China + MIMIC-IV, USA	Retrospective multicohort development/validation	Abdominal surgery; **955**	ICD-10-coded sepsis/severe sepsis/septic shock (development/internal); Sepsis-3 within 30 days (external validation)	Preoperative	Ensemble KNN + SVM; multiple conventional ML models	Train/test + independent validation cohorts
Chen et al., 2025 [2]	China + INSPIRE, South Korea	Multicenter retrospective development + external validation	Intestinal-obstruction surgery; **542**	Sepsis-3 within 7 days after intestinal-obstruction surgery (ΔSOFA ≥ 2 attributed to infection)	Perioperative/end of surgery	SVC, LR, Gaussian NB, GBM, RF, AdaBoost, MLP	Internal validation + 4 external datasets
Fransvea et al., 2026 [3]	Italy, 29 centers	Prospective multicenter development	Acute/emergency surgical patients; **2571**	qSOFA ≥ 2, reported as postoperative sepsis (sepsis-risk proxy)	Preoperative	RF and multiple ML models; ensemble	Nested cross-validation/internal testing
Bunn et al., 2021 [20]	USA	Retrospective national database study	Appendectomy; **223,214**	ACS-NSQIP postoperative sepsis or septic shock (SIRS-based definition)	Preoperative	LR, SVM, RF, XGBoost	Dataset split/internal model comparison
Kamaleswaran et al., 2021 [26]	USA	Retrospective observational pilot	Liver transplantation; **252**	Sepsis-3 onset; dynamic prediction before meeting criteria	Dynamic postoperative, up to 24 h before event	XGBoost using physiological signal features	Repeated 5-fold cross-validation
Chen et al., 2023 [27]	China	Retrospective development + temporal validation	Liver transplantation; **677**	Sepsis-3 within 7 days after liver transplantation (ΔSOFA ≥ 2 consequent to infection)	Pre-/intraoperative	Seven ML models; RF selected	Internal validation + independent temporal cohort
**Broader models including sepsis/septic shock**							
Thottakkara et al., 2016 [24]	USA	Retrospective single-center cohort	Major inpatient surgery; **50,318**	Severe sepsis and other complications	Preoperative	LR, GAM, NB, SVM	70/30 train-validation split
Merath et al., 2020 [28]	USA	Retrospective national database study	Liver, pancreatic, colorectal surgery; **15,657**	Multiple postoperative complications including sepsis/SSI	Pre-/perioperative	Classification/decision trees	Bootstrap/internal validation
Bihorac et al., 2019 [25]	USA	Retrospective development/validation	Major inpatient surgery; **51,457**	Eight major complications including sepsis	Preoperative	MySurgeryRisk ML framework	Internal bootstrap-based validation
Bertsimas et al., 2018 [29]	USA	Retrospective derivation + temporal validation	Emergency surgery; **382,960**	Multiple outcomes including sepsis/septic shock	Preoperative	Optimal Classification Trees (POTTER)	Temporal validation using later NSQIP cohort
El Hechi et al., 2021 [21]	USA	Independent validation study	Emergency general surgery/laparotomy; **59,955**	Multiple outcomes including sepsis/septic shock	Preoperative	POTTER	Independent later NSQIP validation
**Postoperative infection/surgical-site infection**							
Ghabisha et al., 2026 [4]	Yemen	Retrospective cohort	Major gastrointestinal surgery; **525**	SSI	Pre-/intraoperative	LR, RF, XGBoost, neural network	Temporal train/test validation
Hassan et al., 2026 [8]	UK	Retrospective modeling study	Elective general abdominal surgery; **2716**	Postoperative infection	Pre-/perioperative	12 algorithms; gradient boosting/ensemble	Internal cross-validation
Colborn et al., 2023 [30]	USA, 5 hospitals	Retrospective multicenter EHR study	Mixed major surgery; **30,639 operations**	SSI, UTI, sepsis, pneumonia	Postoperative surveillance	Penalized ML models	Chronological held-out test cohort
van der Meijden et al., 2025 [9]	Netherlands + Belgium	Multicenter development, external validation/update	Multiple surgical specialties; **253,010**	7- and 30-day postoperative infection	Pre-/intraoperative	XGBoost/PERISCOPE	External validation, local updating + temporal validation
GlobalSurg Collaborative, 2024 [5]	International	Analysis of prospective international cohorts	Gastrointestinal surgery; **22,483**	30-day SSI	At time of surgery	LASSO, XGBoost	Independent external GlobalSurg cohort
Piebpien et al., 2024 [31]	Thailand	Retrospective validation/model updating	Abdominal surgery; **12,596**	SSI	Perioperative	Updated SENIC-LR, DT, RF, XGBoost, NB	Train/test; external validation/update of SENIC
Azimi et al., 2020 [32]	USA	Retrospective pilot study	Colorectal resection; **208**	Postoperative infection	Pre-/perioperative	DT, SVM, LR, NB, NN, RF	Bootstrap/internal validation
Ohno et al., 2022 [33]	Japan	Retrospective single-center	Colon cancer surgery; **730**	SSI	Perioperative	Prediction One AI	Internal evaluation
Weller et al., 2018 [34]	USA	Retrospective EHR modeling	Colorectal surgery; **9598**	SSI and other postoperative complications	Early postoperative	Multiple statistical/ML classifiers	80/20 random train/test split
Sanger et al., 2016 [19]	USA	Prospective cohort	Open abdominal surgery; **851**	Inpatient SSI	Dynamic postoperative	Naïve Bayes + simplified LR	Training + holdout test cohort
Ke et al., 2017 [35]	USA	Dynamic-data methodological study	Abdominal surgery; **860**	SSI onset/risk	Dynamic postoperative	Spatiotemporal ML + matrix completion	5-fold cross-validation
Sohn et al., 2017 [36]	USA	Retrospective surveillance study	Colorectal surgery; **751**	Clinically important SSI	Postoperative surveillance	Bayesian network + NLP	Internal evaluation
Cho et al., 2024 [37]	South Korea	Retrospective surveillance study	Colon surgery; **1652**	SSI surveillance	Postoperative	LR, RF, GBM, NN ± RFE	Internal training/testing
Celik et al., 2025 [38]	USA	Retrospective development/evaluation	Colon surgery; **1508**	SSI	Postoperative surveillance	LR, RF, XGBoost + NLP features	Stratified 80/20 train-test split
Cobler-Lichter et al., 2025 [22]	USA	Retrospective national trauma database	Emergent trauma laparotomy; **74,806**	Deep/organ-space SSI	Admission/perioperative	ML models + SHAP	Internal train/test modeling
Rahimi et al., 2025 [39]	Iran	Retrospective cohort	Colorectal cancer surgery; **528**	30-day SSI	Pre-/perioperative	RF, KNN, DT, XGBoost	Internal evaluation + imbalance resampling
Al Mamlook et al., 2023 [40]	USA	Retrospective national database study	Broad surgical cohort; **2,882,526**	SSI	Preoperative/procedure factors	LR, NB, RF, DT, SVM, ANN, DNN	10-fold cross-validation
Grass et al., 2021 [41]	USA	Retrospective colorectal cohort	Elective colorectal resection; **2376**	Deep organ-space SSI	Preoperative	Bayesian-Probit MI vs. LR	10-fold CV + cross-institution comparison
Ruan et al., 2022 [42]	USA	Retrospective longitudinal EHR study	Colorectal surgery; **3535**	Multiple postoperative infections	Dynamic/real-time	GRU-D models vs. LR	6-fold CV + held-out test set
**Anastomotic/gastrointestinal leakage**							
Adams & Papagrigoriadis, 2014 [43]	UK	Retrospective development + prospective pilot	Colorectal anastomosis; **76**	Anastomotic leakage	Early postoperative	Genetic artificial neural network	Internal validation + 12-patient prospective pilot
Taha-Mehlitz et al., 2024 [44]	Switzerland	Retrospective multicenter pilot	Colorectal surgery; **1244**	Anastomotic leakage	Preoperative	Multiple ML algorithms; RF/LR highlighted	5-fold CV + external holdout
Ochs et al., 2025 [6]	Europe + North America	Retrospective multicenter prognostic study	Colorectal resection; **9120**	Anastomotic leakage	Pre-/perioperative	Cross-attention meta-model	Cross-validation + external ESCP validation
Martin-Arevalo et al., 2026 [10]	Spain	Development + prospective temporal validation	Minimally invasive colorectal cancer surgery; **1961**	Anastomotic leakage	Within 24 h postoperatively	Five ML architectures; regularized LR selected	Development split + prospective temporal validation
Shao et al., 2021 [45]	China	Retrospective train/test study	Total/proximal gastrectomy; **1660**	Anastomotic leakage	Pre-/intraoperative	LR, RF, SVM, XGBoost	80/20 train-test split
Liu et al., 2022 [46]	China	Retrospective single-center	Gastrectomy for gastric cancer; **297**	Anastomotic leakage	Perioperative/POD4	Five ML models; SVM highlighted	Internal model comparison
van Kooten et al., 2022 [47]	Netherlands	Retrospective nationwide audit	Esophageal/gastric cancer surgery; **6427**	Anastomotic leakage	Pre-/perioperative	GLM, LASSO, KNN, NN, SVM, RF, AdaBoost, SuperLearner	Internal model validation
Nudel et al., 2021 [23]	USA	Retrospective national bariatric database	Weight-loss surgery; **436,807**	Gastrointestinal leak	Preoperative	ANN, XGBoost, LR	Random train/validation/test split
**Postoperative pancreatic fistula**							
Kambakamba et al., 2020 [48]	Switzerland	Retrospective case–control proof-of-principle	Pancreatoduodenectomy; **110**	POPF	Preoperative CT	ML texture analysis	10-fold cross-validation
Lee et al., 2024 [7]	South Korea	Retrospective development + temporal holdout	Pancreatoduodenectomy; **1333**	Any/clinically relevant POPF	Preoperative	Clinical ML + CT deep learning ensemble	Temporal holdout cohort
Han et al., 2020 [49]	South Korea	Retrospective single-center development	Pancreatoduodenectomy; **1769**	POPF	Pre-/intraoperative	RF and neural network ± RFE	Internal evaluation
Shen et al., 2022 [50]	China	Retrospective single-center cohort	Pancreatoduodenectomy; **2421**	Clinically relevant POPF	Dynamic early postoperative	CatBoost, LightGBM, XGBoost, RF	Repeated 5-fold CV + test datasets
Ingwersen et al., 2023 [51]	Netherlands, 16 centers	Retrospective nationwide multicenter audit	Pancreatoduodenectomy; **4912**	Clinically relevant POPF	Pre-/intraoperative	RF, NN, SVM, gradient boosting vs. LR	Bootstrap internal validation
**Other major abdominal postoperative complications**							
Zeng et al., 2021 [52]	China	Retrospective single-center train/test	Liver cancer resection; **175**	Composite postoperative complications	Pre-/intraoperative	LR, C5.0, CART, SVM, RF	70/30 train-test split
Hong et al., 2024 [53]	China, 17 centers	Multicenter development + external validation	Laparoscopic radical gastrectomy; **1396**	Serious 30-day postoperative complications	Pre-/perioperative	LASSO, RF, ANN	998 development + 398 independent external validation

**Abbreviations:** AI, artificial intelligence; ANN, artificial neural network; CART, classification and regression tree; CT, computed tomography; CV, cross-validation; DNN, deep neural network; DT, decision tree; EHR, electronic health record; GAM, generalized additive model; GBM, gradient-boosting machine; GLM, generalized linear model; GRU-D, gated recurrent unit with decay; KNN, k-nearest neighbors; LASSO, least absolute shrinkage and selection operator; LR, logistic regression; MI, multiple imputation; ML, machine learning; MLP, multilayer perceptron; NB, naïve Bayes; NLP, natural-language processing; NN, neural network; POD4, postoperative day 4; POPF, postoperative pancreatic fistula; RF, random forest; RFE, recursive feature elimination; SHAP, SHapley Additive exPlanations; SSI, surgical-site infection; SVC, support-vector classifier; SVM, support-vector machine; UTI, urinary tract infection.

**Table 2:** GRADE-adapted certainty of evidence for predictive performance and transportability of AI/ML models for postoperative sepsis and related complications after major abdominal surgery.

Outcome Domain	Studies, *n*	Risk of Bias	Inconsistency	Indirectness	Imprecision	Publication/Reporting Bias	Overall Certainty	Basis for Judgment
Primary postoperative sepsis/septic shock	6	**Serious**	**Serious**	**Serious**	**Serious**	Not assessable	**VERY LOW**	PROBAST + AI: 3 Low, 2 Unclear, 1 High. Outcomes included Sepsis-3, coded/older criteria, and a qSOFA proxy; prediction tasks and performance varied. Strict Sepsis-3 restriction left four studies/evaluations. Several validation estimates had limited events or wide CIs.
Postoperative infection/SSI	19	**Serious**	**Serious**	Not serious	Not serious	Not assessable	**LOW**	PROBAST + AI: 2 Low, 13 Unclear, 4 High. Infection definitions, timing, clinical tasks, and performance were heterogeneous. However, most evidence directly concerned abdominal/general surgical infection or SSI, and several large external/temporal cohorts yielded reasonably precise estimates.
Anastomotic leakage	8	**Serious**	**Serious**	Not serious	**Serious**	Not assessable	**VERY LOW**	PROBAST + AI: 0 Low, 6 Unclear, 2 High. Performance and validation differed across colorectal, gastric, esophagogastric, and bariatric surgery. Important external/temporal validations were event-limited; Martin-Arevalo had 7 events and AUROC 0.73 (95% CI 0.54–0.92).
Postoperative pancreatic fistula	5	**Serious**	**Serious**	Not serious	**Serious**	Not assessable	**VERY LOW**	PROBAST + AI: 1 Low, 2 Unclear, 2 High. Models differed in fistula definition, predictor timing, data modality, and performance. Independent validation was sparse and clinically relevant fistula estimates were based on limited validation evidence.

**Abbreviations:** AI, artificial intelligence; GRADE, Grading of Recommendations Assessment, Development and Evaluation; ML, machine learning; POPF, postoperative pancreatic fistula; PROBAST + AI, Prediction model Risk Of Bias ASsessment Tool + Artificial Intelligence; SSI, surgical-site infection.

## Data Availability

No new data were created or analyzed in this study. Data sharing is not applicable to this article.

## Supplementary Materials

The following supporting information can be downloaded at: https://www.mdpi.com/article/10.3390/medsci14060685/s1, Table S1, Full reproducible search strategies for PubMed, Scopus, and CENTRAL; Table S2, Study-level clinical-task classification and outcome-definition audit; Table S3, Model-level performance and evaluation data; Table S4, PROBAST + AI signaling-question responses, development-quality, model-evaluation risk-of-bias, and applicability assessments; Table S5, Linked cohorts and narrative sensitivity analyses; PRISMA 2020 Checklist, Completed reporting checklist for the systematic review.

## Abbreviations

The following abbreviations are used in this manuscript:

AI	Artificial intelligence
ANN	Artificial neural network
ASA	American Society of Anesthesiologists
AUC	Area under the curve
AUPRC	Area under the precision–recall curve
AUROC	Area under the receiver operating characteristic curve
BMI	Body mass index
CENTRAL	Cochrane Central Register of Controlled Trials
CI	Confidence interval
CRP	C-reactive protein
CT	Computed tomography
CV	Cross-validation
DNN	Deep neural network
DT	Decision tree
EHR	Electronic health record
GBM	Gradient-boosting machine
GRADE	Grading of Recommendations Assessment, Development and Evaluation
KNN	K-nearest neighbors
LR	Logistic regression
ML	Machine learning
MLP	Multilayer perceptron
NB	Naïve Bayes
NLP	Natural-language processing
NLR	Neutrophil-to-lymphocyte ratio
NN	Neural network
NPV	Negative predictive value
POPF	Postoperative pancreatic fistula
PPV	Positive predictive value
PRISMA	Preferred Reporting Items for Systematic Reviews and Meta-Analyses
PROBAST + AI	Prediction model Risk Of Bias ASsessment Tool + Artificial Intelligence
PROSPERO	International Prospective Register of Systematic Reviews
RF	Random forest
RFE	Recursive feature elimination
ROBINS-I	Risk Of Bias In Non-randomized Studies of Interventions
ROC	Receiver operating characteristic
SHAP	SHapley Additive exPlanations
SSI	Surgical-site infection
SVC	Support-vector classifier
SVM	Support-vector machine
