# Supplementary material for: Before Sepsis Strikes: Artificial Intelligence for Early Prediction of Postoperative Sepsis and Sepsis-Related Complications After Major Abdominal Surgery—A Systematic Review
Source: Med Sci (Basel). 2026 Oct 19;14(6):685. doi: 10.3390/medsci14060685 (PMC13707063; doi:10.3390/medsci14060685)
Supplement: Supplementary file 1 [file medsci-14-00685-s001.zip › Supplementary_Table_S1.pdf]

Supplementary Table S1. Full Reproducible Search Strategies and Benchmark Sensitivity Assessment

Review: Before Sepsis Strikes: Artificial Intelligence for Early Prediction of Postoperative Sepsis and Sepsis-Related Complications After Major Abdominal Surgery - A Systematic Review

Original search date: 10 August 2026 **Coverage:** Database inception to 10 August 2026 **Language restriction:** None **Lower publication-date restriction:** None

Purpose of this supplement. This supplement reproduces the database strategies exactly as performed and recorded in the review protocol. A benchmark term-coverage assessment was additionally undertaken using known eligible studies representing distinct AI/ML model families and clinical applications. The benchmark assessment was used to evaluate whether the absence of explicit labels such as natural-language processing (NLP) or decision tree from parts of the search syntax demonstrated a material terminology gap. The original search history has not been retrospectively rewritten.

Table S1A. Original database searches and yields

Database	Search date	Records retrieved	Search structure	Publication-type filter
PubMed	10 Aug 2026	1,029	AI/ML AND sepsis/related complication AND abdominal/general surgery	Reviews, meta-analyses, editorials, and comments excluded
Scopus	10 Aug 2026	668	AI/ML AND sepsis/related complication AND abdominal/general surgery	Reviews, editorials, and letters excluded by document type
CENTRAL	10 Aug 2026	208	Broader AI/ML AND sepsis/related complication strategy; surgical eligibility applied during screening	No equivalent publication-type filter reported

Total records before deduplication: 1,905.

Table S1B. PubMed - original strategy

Recorded yield: 1,029 records

```
(
"Artificial Intelligence"[Mesh]
OR "Machine Learning"[Mesh]
OR "artificial intelligence"[tiab]
OR "machine learning"[tiab]
OR "deep learning"[tiab]
OR "artificial neural network"[tiab]
OR "neural network"[tiab]
OR "neural networks"[tiab]
OR "random forest"[tiab]
OR "random forests"[tiab]
OR "support vector machine"[tiab]
OR "support vector machines"[tiab]
OR "gradient boosting"[tiab]
OR XGBoost[tiab]
OR CatBoost[tiab]
OR "ensemble learning"[tiab]
OR "Bayesian network"[tiab]
```

```
OR "data mining"[tiab]
)
AND
(
"Sepsis"[Mesh]
OR "Postoperative Complications"[Mesh]
OR "Surgical Wound Infection"[Mesh]
OR sepsis[tiab]
OR "postoperative sepsis"[tiab]
OR "septic shock"[tiab]
OR "postoperative infection"[tiab]
OR "postoperative infections"[tiab]
OR "surgical site infection"[tiab]
OR "surgical site infections"[tiab]
OR "organ space infection"[tiab]
OR "organ space infections"[tiab]
OR "intra-abdominal infection"[tiab]
OR "intra-abdominal infections"[tiab]
OR "anastomotic leak"[tiab]
OR "anastomotic leakage"[tiab]
OR "pancreatic fistula"[tiab]
OR "postoperative complication"[tiab]
OR "postoperative complications"[tiab]
)
AND
(
"Digestive System Surgical Procedures"[Mesh]
OR "General Surgery"[Mesh]
OR "Laparotomy"[Mesh]
OR "abdominal surgery"[tiab]
OR "gastrointestinal surgery"[tiab]
OR "colorectal surgery"[tiab]
OR "colon surgery"[tiab]
OR "rectal surgery"[tiab]
OR gastrectom*[tiab]
OR hepatectom*[tiab]
OR "liver resection"[tiab]
OR pancreaticoduodenectom*[tiab]
OR pancreatectom*[tiab]
OR "bariatric surgery"[tiab]
OR "liver transplantation"[tiab]
OR laparotom*[tiab]
OR appendectom*[tiab]
OR "intestinal obstruction"[tiab]
OR "emergency general surgery"[tiab]
OR "major surgery"[tiab]
OR "emergency surgery"[tiab]
OR "general surgery"[tiab]
)
NOT
(
Review[pt]
OR Meta-Analysis[pt]
OR Editorial[pt]
OR Comment[pt]
```

```
)
```

Table S1C. Scopus - original strategy

Recorded yield: 668 records

```
TITLE-ABS-KEY(
 "artificial intelligence"
 OR "machine learning"
 OR "deep learning"
 OR "artificial neural network"
 OR "neural network"
 OR "random forest"
 OR "support vector machine"
 OR "gradient boosting"
 OR XGBoost
 OR CatBoost
 OR "ensemble learning"
 OR "Bayesian network"
 OR "data mining"
)
AND
TITLE-ABS-KEY(
 sepsis
 OR "postoperative sepsis"
 OR "septic shock"
 OR "postoperative infection"
 OR "surgical site infection"
 OR "organ space infection"
 OR "intra-abdominal infection"
 OR "anastomotic leak"
 OR "anastomotic leakage"
 OR "pancreatic fistula"
 OR "postoperative complication"
)
AND
TITLE-ABS-KEY(
 "abdominal surgery"
 OR "gastrointestinal surgery"
 OR "colorectal surgery"
 OR "colon surgery"
 OR "rectal surgery"
 OR gastrectomy
 OR hepatectomy
 OR "liver resection"
 OR pancreaticoduodenectomy
 OR pancreatectomy
 OR "bariatric surgery"
 OR "liver transplantation"
 OR laparotomy
 OR appendectomy
 OR "intestinal obstruction"
 OR "emergency general surgery"
```

```
 OR "major surgery"
 OR "emergency surgery"
 OR "general surgery"
)
AND NOT DOCTYPE(re)
AND NOT DOCTYPE(ed)
AND NOT DOCTYPE(le)
```

Table S1D. CENTRAL - original strategy

Recorded yield: 208 records

```
(("artificial intelligence" OR "machine learning" OR "deep learning" OR "neural network*" OR "artificial neural network*" OR "random forest*" OR "support vector machine*" OR "gradient boosting" OR XGBoost OR CatBoost OR "ensemble learning" OR "Bayesian network*" OR "data mining" OR "decision tree*"):ti,ab,kw)
AND
((sepsis OR "septic shock" OR "postoperative sepsis" OR "post-operative sepsis" OR "postoperative infection*" OR "post-operative infection*" OR "surgical site infection*" OR "surgical-site infection*" OR "organ space infection*" OR "intra-abdominal infection*" OR "anastomotic leak*" OR "anastomotic leakage" OR "pancreatic fistula*" OR "postoperative pancreatic fistula*" OR "postoperative complication*" OR "post-operative complication*"):ti,ab,kw)
```

Table S1E. Benchmark sensitivity assessment of the original strategy

Benchmark method. A purposive benchmark set of known eligible studies was selected to represent distinct model families, including NLP-assisted surveillance, Bayesian networks, decision/classification-tree methods, and other machine-learning applications. The assessment examined whether each benchmark report contained at least one AI/ML concept already present in the original strategy together with relevant outcome and surgical-population terminology. This was used to assess term coverage without retrospectively altering the original search history.

Benchmark study	Methodological feature relevant to audit	Method-specific label explicit in original AI block?	Existing original term(s) providing coverage	Assessment
Sohn et al. [36]	Bayesian network + NLP for colorectal SSI detection	NLP: No; Bayesian network: Yes	"Bayesian network" + surgical-site-infection + colorectal-surgery concepts	Term coverage present
Merath et al. [28]	Decision/classification-tree modeling of postoperative complications	Decision tree: No in PubMed/Scopus	"machine learning" + postoperative-complication + liver/pancreatic/colorectal-surgery concepts	Term coverage present
Cho et al. [37]	Machine-learning SSI surveillance using RF/GBM/NN approaches	Core ML term: Yes	"machine learning" + surgical-site-infection + colon-surgery concepts	Term coverage present
Celik et al. [38]	Machine learning + NLP for colon-SSI surveillance	NLP: No; RF/XGBoost families: Yes	"machine learning" / "random forest" / XGBoost + SSI + colon-surgery concepts	Term coverage present
Bertsimas et al. [29]	Machine-learning classification-tree framework (POTTER)	Classification tree: No	"machine learning" + emergency/general-surgery + postoperative-complication concepts	Term coverage present

Interpretation. The benchmark assessment did not identify a demonstrable terminology gap within the selected known eligible evidence: representative studies using NLP, Bayesian networks, decision/classification-tree methods, and related machine-learning approaches contained at least one broader AI/ML concept already represented in the original search, together with relevant outcome and surgical terminology. CENTRAL additionally included “decision tree*” explicitly. Accordingly, the original database strings are retained and reported as actually performed rather than

retrospectively reconstructed. The absence of every individual algorithm label from the PubMed and Scopus AI blocks is therefore interpreted as a potential search-term limitation, but not as evidence that the benchmark studies were unretrievable.

Table S1F. Database-selection and publication-type-filter rationale

Component	Rationale	Interpretation / qualification
PubMed	Selected as the principal biomedical database because it covers clinical surgery, perioperative medicine, infectious complications, and biomedical informatics.	Retained as a core biomedical source.
Scopus	Selected as a complementary multidisciplinary source with broad clinical, surgical, technical/informatics, and citation-indexing coverage.	Used to complement rather than duplicate PubMed coverage.
CENTRAL	Included as a complementary high-sensitivity source despite prediction-model evidence being predominantly observational. The protocol intentionally used a broader AI/ML-plus-outcome strategy and applied abdominal/general-surgical eligibility during screening.	Its trial-focused scope was recognized; it functioned as a supplementary rather than primary source.
Backward/forward citation searching	Used to supplement database retrieval and reduce dependence on any single indexing vocabulary.	Route-specific historical records documenting the exact citation-search platform, date, and number of records generated were not retained in sufficient detail to permit reliable retrospective reconstruction.
Scopus letter filter	The original Scopus strategy excluded the document type “letter” as a precision filter. The review-level protocol, however, excluded letters only when they lacked sufficient original data.	This database-specific filter is acknowledged as narrower than the review-level eligibility wording. PubMed and CENTRAL did not apply an equivalent categorical letter exclusion, and eligibility was ultimately based on study content. No benchmark study was identified as being lost because of this filter.

Overall search-sensitivity conclusion

The full database strategies and recorded yields are reported above exactly as documented in the protocol. The benchmark assessment was used to test whether representative NLP, Bayesian-network, decision/classification-tree, and related machine-learning studies depended on method labels absent from the original PubMed/Scopus strings. In the selected benchmark set, broader AI/ML concepts already present in the original strategy provided a term-level retrieval route for each study, and CENTRAL already contained “decision tree*”. Therefore, no retrospective replacement of the original strategy was undertaken. The database selection and the Scopus publication-type filter are now explicitly justified and qualified.

Note: Reference numbers correspond to the main manuscript. The retained review records permitted verification of the database search strategies, database yields, deduplication counts, screening totals, aggregate full-text exclusion categories, and final study count. Historical route-specific records sufficient to reconstruct the exact citation-search platform/date and counts, the identities of the three unretrieved reports, all 90 individual full-text exclusions, or a complete author-contact log were not available in the retained review records. These details were therefore not inferred or retrospectively reconstructed.
