# Supplementary material for: Before Sepsis Strikes: Artificial Intelligence for Early Prediction of Postoperative Sepsis and Sepsis-Related Complications After Major Abdominal Surgery—A Systematic Review
Source: Med Sci (Basel). 2026 Oct 19;14(6):685. doi: 10.3390/medsci14060685 (PMC13707063; doi:10.3390/medsci14060685)
Supplement: Supplementary file 1 [file medsci-14-00685-s001.zip › Supplementary_Table_S2.pdf]

Supplementary Table S2. Clinical Task and Outcome-Definition Audit of Included Studies

Before Sepsis Strikes: Artificial Intelligence for Early Prediction of Postoperative Sepsis and Sepsis-Related Complications After Major Abdominal Surgery — A Systematic Review

Purpose and interpretation. This supplement documents the clinical task, predictor timing, target-population applicability, outcome definition, ascertainment method, outcome window, and demonstrated lead time for each included study. Classification was based on the temporal ordering between predictor availability and outcome ascertainment rather than on labels such as "preoperative" or "postoperative" alone. Score-based proxies, administrative/coded outcomes, consensus-defined clinical outcomes, and retrospective surveillance tasks were not treated as interchangeable.

Abbreviations and conventions. NR, not reported or not sufficiently specified in the main primary report; CDC, Centers for Disease Control and Prevention; NHSN, National Healthcare Safety Network; ACS-NSQIP, American College of Surgeons National Surgical Quality Improvement Program; SOFA, Sequential Organ Failure Assessment; qSOFA, quick SOFA; SSI, surgical-site infection; AL, anastomotic leakage; POPF, postoperative pancreatic fistula; CR-POPF, clinically relevant POPF; ISGPS, International Study Group of Pancreatic Surgery. "PDF p." refers to the page number of the primary-study PDF used for extraction.

Table S2A. Clinical-task, timing, and target-population applicability audit

Study [ref]	Surgical population	Abdominal representation	Abdominal-specific performance?	Evidence role	Clinical task	Prediction/index time & latest predictor	Intended use	Outcome window	Demonstrated lead time
Shu et al., 2025 [1]	Abdominal surgery; development/internal cohorts plus selected MIMIC-IV surgical validation cohort	100% target abdominal-surgical cohorts	Yes	Principal	Future-event prognostic prediction	Preoperative; latest predictors before surgery	Preoperative sepsis-risk stratification	Development: postoperative; external validation: within 30 d	NR
Chen et al., 2025 (intestinal obstruction) [2]	Intestinal-obstruction surgery	100%	Yes	Principal	Future-event prognostic prediction	Perioperative/end of surgery	Postoperative decision support after IO surgery	Within 7 d	NR
Fransvea et al., 2026 [3]	Acute/emergency surgery across abdominal, thoracic, vascular, gynecologic, and urologic procedures	Mixed; abdominal proportion not separately reported in main article	No	Contextual-indirect for abdominal-specific performance	Future-event prognostic prediction	Preoperative only	Emergency-surgery risk stratification	NR	NR
Bunn et al., 2021 [20]	Appendectomy	100%	Yes	Principal	Future-event prognostic prediction	Preoperative	Preoperative screening for postoperative sepsis/septic shock	ACS-NSQIP postoperative window (30 d)	NR
Kamaleswaran et al., 2021 [26]	Liver transplantation ICU cohort	100%	Yes	Principal	Early detection / dynamic monitoring	Continuous postoperative physiology; up to 24 h before Sepsis-3 alert timestamp	Dynamic early warning and escalation	Event-based	12 h performance explicitly reported
Chen et al., 2023 (liver transplantation) [27]	Liver transplantation	100%	Yes	Principal	Future-event prognostic prediction	Pre-/intraoperative; prediction after surgery using perioperative variables	Early postoperative sepsis-risk stratification	Within 7 d	NR
Thottakkara et al., 2016 [24]	Major inpatient surgery, multiple surgical specialties	Mixed; abdominal proportion NR	No	Contextual-indirect	Future-event prognostic prediction	Preoperative	Preoperative risk forecasting	Severe sepsis at any time after surgery	NR
Merath et al., 2020 [28]	Liver, pancreatic, and colorectal surgery	100% abdominal/GI	Yes	Contextual (broader multi-outcome model)	Future-event prognostic prediction	Pre-/perioperative	Risk stratification for multiple complications	30 d	NR
Bihorac et al., 2019 (MySurgeryRisk) [25]	Major inpatient surgery, multiple surgical specialties	Mixed; abdominal proportion NR	No	Contextual-indirect	Future-event prognostic prediction	Preoperative	Automated preoperative risk stratification	30 postoperative d	NR
Bertsimas et al., 2018 (POTTER derivation) [29]	Emergency surgery across ACS-NSQIP procedures	Mixed emergency-surgery population; abdominal proportion NR	Not for full cohort	Contextual-indirect; abdominal relevance through EGS procedures	Future-event prognostic prediction	Preoperative	Emergency-surgery risk counseling/triage	30 d	NR

Study [ref]	Surgical population	Abdominal representation	Abdominal-specific performance?	Evidence role	Clinical task	Prediction/index time & latest predictor	Intended use	Outcome window	Demonstrated lead time
El Hechi et al., 2021 (POTTER validation) [21]	Emergency general surgery; emergency-laparotomy subgroup	Mixed EGS overall; emergency-laparotomy subgroup directly abdominal	Yes for laparotomy subgroup	Principal for emergency-laparotomy subgroup; contextual for full EGS cohort	Future-event prognostic prediction	Preoperative	Emergency postoperative triage/risk estimation	30 d	NR
Ghabisha et al., 2026 [4]	Major gastrointestinal surgery	100%	Yes	Principal	Future-event prognostic prediction	Pre-/intraoperative	SSI prevention/risk stratification	Within 30 d	NR
Hassan et al., 2026 (SMART) [8]	Elective general abdominal surgery	100%	Yes	Principal	Future-event prediction (intended); temporal-order concern	Intended preoperative prediction; source dataset also contained hospitalization/discharge variables, so latest permissible predictor time is not fully clear	Preoperative infection-risk reduction	Postoperative infection after admission; patients with infection at admission/within first 48 h excluded	NR
Colborn et al., 2023 [30]	Mixed major surgery across 5 hospitals	Mixed; abdominal proportion NR	No	Contextual-indirect	Retrospective case ascertainment / surveillance	Postoperative EHR data used for surveillance; variables could accrue during 30-d period	Automated infection surveillance/reporting	Within 30 d	Not a pre-event lead-time study
van der Meijden et al., 2025 (PERISCOPE) [9]	Multiple surgical specialties across 3 hospitals	Mixed; abdominal proportion NR	No clearly separable abdominal-specific headline performance	Contextual-indirect	Future-event prognostic prediction	Preoperative + intraoperative data only	Early risk prediction, discharge support, timely treatment	7 d and 30 d	NR
GlobalSurg Collaborative, 2024 (GloSSI) [5]	Gastrointestinal surgery	100%	Yes	Principal	Future-event prognostic prediction	At time of surgery	30-day SSI risk stratification	30 d	NR
Piebpien et al., 2024 [31]	Abdominal surgery	100%	Yes	Principal	Future-event prognostic prediction	Perioperative	SSI risk stratification/model updating	Within 30 d	NR
Azimi et al., 2020 [32]	Colorectal resection	100%	Yes	Principal with temporal-order caveat	Future-event prediction (intended); predictor leakage concern	Predictor set included variables such as length of stay/discharge destination in some analyses, so latest predictor time may extend postoperatively	Identify patients likely to develop postoperative infection	NR	NR
Ohno et al., 2022 [33]	Colon cancer surgery	100%	Yes	Principal	Future-event prognostic prediction	Perioperative	SSI risk prediction	NR	NR
Weller et al., 2018 [34]	Colorectal surgery	100%	Yes	Principal	Sequential prediction / early postoperative monitoring	Preoperative through POD2	Updating postoperative complication risk	30 d	Time-specific predictions through POD2; event-specific lead NR
Sanger et al., 2016 [19]	Open abdominal surgery	100%	Yes	Principal	Early detection / dynamic monitoring	Baseline plus daily wound/vital-sign features from POD2	Early identification of evolving inpatient SSI	30-d ascertainment; model analysis focused on inpatient SSI	Features discernible before diagnosis; exact lead varied
Ke et al., 2017 [35]	Open abdominal surgery cohort	100%	Yes	Principal	Early detection / dynamic monitoring	Daily data from POD2 to discharge or 21 d	Predict SSI onset time from dynamic health data	Observation to discharge/21 d; 30-d follow-up for post-discharge infection	Model predicts days to onset; exact clinically useful lead varies
Sohn et al., 2017 [36]	Colorectal surgery	100%	Yes	Contextual surveillance evidence	Retrospective case ascertainment / surveillance	Postoperative chart/text features across 30-d period	Automated identification of clinically important SSI	Within 30 d	No demonstrated pre-event lead time
Cho et al., 2024 [37]	Colon surgery	100%	Yes	Contextual surveillance evidence	Retrospective case ascertainment / surveillance	Postoperative administrative, laboratory, microbiology, readmission/reoperation and treatment variables	Reduce manual SSI surveillance workload	NHSN surveillance window	No pre-event lead time demonstrated

Study [ref]	Surgical population	Abdominal representation	Abdominal-specific performance?	Evidence role	Clinical task	Prediction/index time & latest predictor	Intended use	Outcome window	Demonstrated lead time
Celik et al., 2025 [38]	Colon surgery	100%	Yes	Contextual surveillance evidence	Retrospective case ascertainment / surveillance	Postoperative structured EHR + clinical-note NLP	Automated SSI surveillance/case finding	30 d	No pre-event lead time demonstrated
Cobler-Lichter et al., 2025 [22]	Emergent trauma laparotomy	100%	Yes	Principal	Future-event prognostic prediction	Admission/perioperative	Risk prediction of clinically meaningful SSI	In-hospital primary outcome	NR
Rahimi et al., 2025 [39]	Colorectal cancer surgery	100%	Yes	Principal	Future-event prognostic prediction	Pre-/perioperative	30-day SSI risk stratification	Up to 30 d	NR
Al Mamlook et al., 2023 [40]	Broad ACS-NSQIP surgical cohort (general, vascular and other specialties)	Mixed; abdominal proportion NR	No	Contextual-indirect	Future-event prognostic prediction	Preoperative/procedure factors	SSI risk prediction	30 d	NR
Grass et al., 2021 [41]	Elective colorectal resection	100%	Yes	Principal	Future-event prognostic prediction	Preoperative	Deep/organ-space colorectal SSI risk prediction	NSQIP postoperative window	NR
Ruan et al., 2022 [42]	Colorectal surgery	100%	Yes	Principal	Dynamic/real-time prediction	Longitudinal EHR updated over postoperative course	Real-time risk prediction for post-surgical complications	Within 30 d	Time-updated; exact event-specific lead varies
Adams & Papagrigoriadis, 2014 [43]	Colorectal anastomosis	100%	Yes	Principal	Early detection	Early postoperative clinical/laboratory features	Earlier recognition of clinical anastomotic leak	During postoperative recovery	Intended early detection; exact hours/days NR
Taha-Mehlitz et al., 2024 [44]	Colorectal surgery with bowel anastomosis	100%	Yes	Principal	Future-event prognostic prediction	Preoperative	Preoperative AL risk prediction	NR	NR
Ochs et al., 2025 [6]	Colorectal resection	100%	Yes	Principal	Future-event prognostic prediction	Pre-/perioperative	Prediction of AL incidence	NR	NR
Martin-Arevalo et al., 2026 [10]	Minimally invasive colorectal cancer surgery	100%	Yes	Principal	Early detection / postoperative prediction	Within 24 h postoperatively	Early AL detection to guide postoperative management	Prospective temporal validation; event follow-up per study protocol	Prediction at 24 h; exact time-to-clinical-diagnosis lead not uniformly reported
Shao et al., 2021 [45]	Total/proximal gastrectomy	100%	Yes	Principal	Future-event prognostic prediction	Pre-/intraoperative	AL risk prediction	Postoperative follow-up NR in main extraction	NR
Liu et al., 2022 [46]	Gastrectomy for gastric cancer	100%	Yes	Principal	Early postoperative prediction / detection	Perioperative through POD4	Early identification of postoperative AL	Postoperative	By POD4; exact lead to diagnosis NR
van Kooten et al., 2022 [47]	Esophageal and gastric cancer surgery	100%	Yes	Principal	Future-event prognostic prediction	Pre-/perioperative	AL risk prediction	Postoperative	NR
Nudel et al., 2021 [23]	Weight-loss/bariatric surgery	100%	Yes	Principal	Future-event prognostic prediction	Preoperative	30-day gastrointestinal-leak risk prediction	30 d	NR
Kambakamba et al., 2020 [48]	Pancreatoduodenectomy	100%	Yes	Principal	Future-event prognostic prediction	Preoperative CT	Preoperative POPF risk prediction	Postoperative	NR
Lee et al., 2024 [7]	Pancreatoduodenectomy	100%	Yes	Principal	Future-event prognostic prediction	Preoperative CT + clinical data	Preoperative POPF/CR-POPF risk stratification	Postoperative	NR
Han et al., 2020 [49]	Pancreatoduodenectomy	100%	Yes	Principal	Future-event prognostic prediction	Pre-/intraoperative	POPF risk prediction	Postoperative	NR
Shen et al., 2022 [50]	Pancreatoduodenectomy	100%	Yes	Principal	Early diagnosis / dynamic monitoring	Pre/intraoperative + serial postoperative laboratory/drain data	Early CR-POPF diagnosis and drain-removal guidance	Dynamic postoperative	Increasing information over postoperative days; exact lead NR
Ingwersen et al., 2023 [51]	Pancreatoduodenectomy	100%	Yes	Principal	Future-event prognostic prediction	Pre-/intraoperative	POPF and delayed-gastric-emptying risk prediction	Postoperative	NR
Zeng et al., 2021 [52]	Liver resection for liver cancer	100%	Yes	Contextual broader-complication evidence	Future-event prognostic prediction	Pre-/intraoperative	Risk of composite postoperative complications	Postoperative; exact window NR	NR

Study [ref]	Surgical population	Abdominal representation	Abdominal-specific performance?	Evidence role	Clinical task	Prediction/index time & latest predictor	Intended use	Outcome window	Demonstrated lead time
Hong et al., 2024 [53]	Laparoscopic radical gastrectomy	100%	Yes	Contextual broader-complication evidence	Future-event prognostic prediction	Pre-/perioperative	30-day serious-complication risk prediction	Within 30 d	NR

Table S2B. Study-level outcome-definition and ascertainment audit

Study [ref]	Exact modeled outcome / definition used in review	Ascertainment / important qualification	Outcome window	Primary-source location
Shu et al., 2025 [1]	Development/internal: postoperative sepsis defined by ICD-10 diagnosis of sepsis, severe sepsis, or septic shock. External validation: Sepsis-3 after surgery within 30 d.	External validation included manual checking after data extraction; preoperative sepsis/infection excluded.	Development: postoperative; external validation: within 30 d	Methods, PDF p.3
Chen et al., 2025 (intestinal obstruction) [2]	Sepsis-3; daily SOFA manually reviewed. Baseline SOFA = day of surgery; acute increase >=2 points attributed to infection defined organ dysfunction.	Electronic medical record review; preoperative sepsis excluded.	Within 7 d	Methods, PDF p.4
Fransvea et al., 2026 [3]	Binary modeled endpoint was qSOFA >=2 vs <2, although authors described the target as postoperative sepsis and referenced Sepsis-3.	Score-based proxy; infection and SOFA-defined organ dysfunction were not independently required by the modeled endpoint.	NR	Methods, PDF p.6
Bunn et al., 2021 [20]	ACS-NSQIP postoperative sepsis/septic shock using an older SIRS-based definition; septic shock required sepsis plus documented organ/circulatory dysfunction.	NSQIP-coded outcome; sepsis in the 48 h before appendectomy was excluded.	ACS-NSQIP postoperative window (30 d)	Methods, PDF p.2
Kamaleswaran et al., 2021 [26]	Sepsis onset identified using Sepsis-3 as the reference event timestamp.	Serial ICU data anchored to Sepsis-3 event; exact infection-adjudication process beyond Sepsis-3 implementation not fully detailed in main text.	Event-based	Abstract/Methods, PDF pp.1-2
Chen et al., 2023 (liver transplantation) [27]	Sepsis-3; daily SOFA manually checked. Baseline SOFA = day of operation; acute total SOFA increase >=2 consequent to infection defined organ dysfunction.	Electronic medical record review; preoperative sepsis excluded.	Within 7 d	Methods, PDF p.3
Thottakkara et al., 2016 [24]	Severe sepsis derived from AHRQ postoperative-sepsis criteria, with ICD-9-CM acute organ-dysfunction codes added to identify organ failure associated with sepsis.	Administrative/EHR-coded outcome.	Severe sepsis at any time after surgery	Methods, PDF p.3
Merath et al., 2020 [28]	ACS-NSQIP postoperative outcomes, including sepsis, septic shock, superficial/deep/organ-space SSI and other complications.	Registry-defined outcomes; individual NSQIP definitions not reproduced in full in the main article.	30 d	Methods, PDF p.2
Bihorac et al., 2019 (MySurgeryRisk) [25]	Eight postoperative complications including sepsis; sepsis reference standard based on administrative codes rather than contemporary consensus sepsis definitions.	Institutional EHR/administrative coding.	30 postoperative d	Methods/Limitations, PDF pp.2,8-9
Bertsimas et al., 2018 (POTTER derivation) [29]	ACS-NSQIP 30-day mortality, morbidity, and 18 individual postoperative complications, including sepsis, septic shock, SSI and organ-space SSI.	Registry-defined outcomes.	30 d	Methods, PDF p.2
El Hechi et al., 2021 (POTTER validation) [21]	ACS-NSQIP postoperative complications, including sepsis and septic shock, validated using the existing POTTER model.	Registry-defined outcomes; laparotomy subgroup separately reported.	30 d	Methods/Results, PDF pp.3-4
Ghabisha et al., 2026 [4]	SSI according to CDC/NHSN criteria; primary article specifies purulent drainage, positive wound cultures, clinical signs of infection and related criteria.	Independent chart review used for a subset; temporal test cohort.	Within 30 d	Methods, PDF p.3
Hassan et al., 2026 (SMART) [8]	Custom bacterial postoperative-infection definition: abnormal temperature (<36 or >38 C) AND abnormal leukocyte count (<4,000 or >11,000) AND at least one additional microbiologic/clinical infection criterion specified by authors.	Hospital EHR/microbiology; not a standard SSI-only endpoint.	Postoperative infection after admission; patients with infection at admission/within first 48 h excluded	Methods, PDF p.2
Colborn et al., 2023 [30]	Composite postoperative infection surveillance: SSI, UTI, sepsis, and pneumonia within 30 d, linked to ACS-NSQIP outcomes.	Structured EHR linked to ACS-NSQIP reference outcomes.	Within 30 d	Abstract/Methods, PDF pp.1-2
van der Meijden et al., 2025 (PERISCOPE) [9]	Overall postoperative infection within 7 or 30 d; site-specific EHR outcome definitions harmonized for model development/validation.	Retrospective EHR outcome ascertainment across hospitals; local updating and temporal validation.	7 d and 30 d	Abstract/Methods, PDF pp.1-3
GlobalSurg Collaborative, 2024 (GloSSI) [5]	CDC-defined SSI including superficial, deep, and organ-space infection. Events recorded at 30 d by in-person or health-record review; where 30-d follow-up was infeasible, SSI at discharge was used pragmatically.	Prospective international cohorts.	30 d	Methods, PDF p.2
Piebpien et al., 2024 [31]	SSI within 30 d identified from infection-control nurse records and electronic hospital databases; diagnosis/procedure/antibiotic/laboratory follow-up data used.	Hospital surveillance and electronic database ascertainment.	Within 30 d	Methods, PDF p.3
Azimi et al., 2020 [32]	Postoperative infection/SSI status from institutional EHR; detailed diagnostic criteria were not explicitly reproduced in the main report.	Retrospective institutional EHR.	NR	Methods, PDF pp.2-3
Ohno et al., 2022 [33]	SSI included superficial and deep incisional infections based on CDC criteria plus wound infection characterized by purulent fluid/pus and local inflammatory signs.	Clinical record-based ascertainment.	NR	Methods, PDF p.2
Weller et al., 2018 [34]	SSI among prespecified postoperative complications; exact SSI diagnostic criteria were not fully reproduced in the main article.	Institutional EHR/NSQIP-linked postoperative outcome data.	30 d	Methods/Results, PDF pp.2,8
Sanger et al., 2016 [19]	CDC criteria for superficial, deep and/or organ-space SSI. Follow-up at POD30 by clinic/telephone/letter; charts, discharge letters, wound photos, and cultures reviewed after discharge.	For analysis, SSI group = any of 3 SSI types; non-SSI group = no inpatient SSI; post-discharge SSI did not disqualify controls.	30-d ascertainment; model analysis focused on inpatient SSI	Methods, PDF pp.8-9
Ke et al., 2017 [35]	Same prospective dynamic-wound cohort framework; SSI defined using CDC criteria, with daily observation. Analysis focused on SSI developing during the observation period.	30-d follow-up by clinic/phone/letter; first SSI used when multiple occurred.	Observation to discharge/21 d; 30-d follow-up for post-discharge infection	Methods, PDF pp.6-7
Sohn et al., 2017 [36]	Clinically important colorectal SSI (CM-SSI) occurring within 30 d; case-identification framework derived from clinical records rather than prospective pre-event forecasting.	Retrospective record review / Bayesian network + NLP.	Within 30 d	Abstract/Methods, PDF pp.3-4
Cho et al., 2024 [37]	Colon SSI confirmed using conventional infection-prevention surveillance according to CDC/NHSN criteria.	Infection-prevention surveillance; model uses postoperative signals such as LOS, readmission, reoperation, cultures, CRP, imaging/procedures and antibiotics.	NHSN surveillance window	Methods, PDF pp.2-4

Study [ref]	Exact modeled outcome / definition used in review	Ascertainment / important qualification	Outcome window	Primary-source location
Celik et al., 2025 [38]	SSI labels assigned by infection-prevention specialists according to CDC/NHSN definitions.	Daily manual IPC review of charts, microbiology, wound assessments and nursing notes; ambiguous cases resolved by consensus; minimum 30-d follow-up.	30 d	Methods, PDF pp.2-3
Cobler-Lichter et al., 2025 [22]	Clinically meaningful SSI diagnosed in hospital, defined as a composite including deep and/or organ-space SSI.	Trauma registry/clinical outcome ascertainment; superficial SSI excluded from primary composite.	In-hospital primary outcome	Methods, PDF p.2
Rahimi et al., 2025 [39]	CDC-defined SSI: superficial, deep and organ-space categories. Endpoint included occurrence of any of the three within 30 d.	In-hospital plus scheduled post-discharge follow-up through 30 d; severity also described with Clavien-Dindo.	Up to 30 d	Methods, PDF p.2
Al Mamlook et al., 2023 [40]	Binary ACS-NSQIP SSI outcome; article relies on NSQIP outcome coding and does not reproduce all subtype diagnostic criteria in the main text.	National registry outcome.	30 d	Methods, PDF pp.2-3
Grass et al., 2021 [41]	Deep or organ-space colorectal SSI according to CDC National Nosocomial Infection Surveillance criteria; infection involving organs/spaces other than superficial incisional compartment.	Institutional ACS-NSQIP cohort; first outcome defined as C-OSI.	NSQIP postoperative window	Methods, PDF p.2
Ruan et al., 2022 [42]	NSQIP-adjudicated postoperative complications within 30 d, including deep/organ-space SSI and other infection-related outcomes.	EHR + NSQIP outcome adjudication; dynamic GRU-D architecture.	Within 30 d	Abstract/Methods, PDF pp.1-3
Adams & Papagrigoriadis, 2014 [43]	Clinical anastomotic leak confirmed at reoperation; controls had delayed recovery but leak was excluded and no reoperation was required.	Clinical/reoperative confirmation.	During postoperative recovery	Abstract, PDF p.1
Taha-Mehlitz et al., 2024 [44]	AL defined according to Rahbari/International Study Group concept as a defect at the anastomotic site creating communication between intra- and extraluminal compartments.	Clinical multicenter records; exact diagnostic ascertainment pathway not fully detailed in abstract.	NR	Introduction/Methods, PDF p.1
Ochs et al., 2025 [6]	AL defined using the International Study Group of Rectal Cancer concept: defect in intestinal wall at the anastomotic site resulting in communication between intraluminal and extraluminal space.	Multicenter clinical datasets; model-development outcome.	NR	Methods/Introduction, PDF p.2
Martin-Arevalo et al., 2026 [10]	Anastomotic leakage diagnosed according to the study's colorectal clinical criteria; article references International Study Group definitions. Exact operational diagnostic components should be read with the primary report.	Prospective temporal validation cohort; outcome clinically adjudicated in surgical follow-up.	Prospective temporal validation; event follow-up per study protocol	Methods, PDF pp.3-4
Shao et al., 2021 [45]	Postoperative anastomotic leakage after gastrectomy; diagnostic definition not reproduced in sufficient detail in the main-text extraction used for this audit.	Clinical record ascertainment.	Postoperative follow-up NR in main extraction	Methods, PDF pp.2-3
Liu et al., 2022 [46]	Postoperative anastomotic leakage after gastric surgery; detailed operational diagnostic criteria were not fully reproduced in the main report text available for extraction.	Clinical record outcome.	Postoperative	Methods, PDF pp.2-3
van Kooten et al., 2022 [47]	Anastomotic leakage defined as any clinically or radiologically proven anastomotic leakage.	Clinical and/or radiologic confirmation.	Postoperative	Methods, PDF p.4
Nudel et al., 2021 [23]	Composite MBSAQIP leak endpoint: postoperative organ-space infection, drain present >30 d, or leak suspected as reason for readmission, reintervention, or reoperation.	MBSAQIP registry outcome.	30 d	Methods, PDF p.2
Kambakamba et al., 2020 [48]	POPF assessed using International Study Group fistula definitions; analytic set included biochemical leak and clinically relevant grade B/C events, with most events grade B/C.	Retrospective case-control sample (55 POPF/55 controls); underlying cohort prevalence differs from analytic sample.	Postoperative	Methods/Results, PDF pp.3-4
Lee et al., 2024 [7]	POPF defined according to International Study Group in Pancreatic Surgery; grades B and C classified as clinically relevant POPF (CR-POPF).	Clinical outcome from institutional cohort; both all POPF and CR-POPF modeled.	Postoperative	Methods, PDF p.7
Han et al., 2020 [49]	2016 ISGPS definition and grading of POPF; grade A removed from POPF classification.	Institutional prospective database retrospectively analyzed; postoperative drain/CT monitoring described.	Postoperative	Methods, PDF p.7
Shen et al., 2022 [50]	CR-POPF according to updated ISGPS 2016 guidelines, grades B/C only. Grade B required POPF plus management consequences; grade C included reoperation, organ failure or death.	Institutional clinical outcome; detailed grade B/C criteria explicitly reported.	Dynamic postoperative	Methods, PDF p.2
Ingwersen et al., 2023 [51]	POPF and DGE defined by International Study Group of Pancreatic Surgery; only grade B/C POPF and DGE were included.	Dutch Pancreatic Cancer Audit; registry-defined outcomes.	Postoperative	Methods, PDF p.2
Zeng et al., 2021 [52]	Composite postoperative complications after liver resection. Article discusses ISGLS-defined liver-surgery complications (hemorrhage, liver failure, bile leakage) but the exact modeled composite operational definition is incompletely specified in the main report.	Clinical record outcome; interpret as broad composite rather than sepsis-specific endpoint.	Postoperative; exact window NR	Methods/Introduction, PDF pp.1-3
Hong et al., 2024 [53]	Thirty-day postoperative complications graded by Clavien-Dindo; grade >=II defined as serious. Composite included infectious and noninfectious complications.	Multicenter clinical outcome; independent external validation cohort.	Within 30 d	Methods, PDF pp.2-3

Table S2C. Focused audit of the six primary sepsis-related studies

Study	Outcome terminology/criteria	Infection required?	Organ dysfunction required?	qSOFA proxy?	Pre-existing sepsis excluded?	Ascertainment / qualification	Source
Shu et al. [1]	Development/internal: ICD-10 sepsis/severe sepsis/septic shock; external: Sepsis-3 within 30 d	External Sepsis-3: Yes; development coding: not independently verifiable	External Sepsis-3: Yes; development coding: depends on coded diagnosis	No	Yes	Development coded outcome; external manual checking	Methods, PDF p.3
Chen et al. [2]	Sepsis-3 within 7 d after IO surgery	Yes	Yes; Delta SOFA >=2 attributed to infection	No	Yes	Daily SOFA manually reviewed in EMR	Methods, PDF p.4
Fransvea et al. [3]	qSOFA >=2 vs <2, reported by authors as postoperative sepsis	No explicit infection requirement in modeled endpoint	No SOFA-based organ-dysfunction requirement in modeled endpoint	Yes	Unclear	Score-based proxy; not independently established Sepsis-3	Methods, PDF p.6

Study	Outcome terminology/criteria	Infection required?	Organ dysfunction required?	qSOFA proxy?	Pre-existing sepsis excluded?	Ascertainment / qualification	Source
Bunn et al. [20]	ACS-NSQIP postoperative sepsis or septic shock, older SIRS-based definition	Infection/end-organ ischemia component reported	Septic shock required organ/circulatory dysfunction; sepsis used older SIRS framework	No	Yes; preoperative sepsis within 48 h excluded	Registry definition	Methods, PDF p.2
Kamaleswaran et al. [26]	Sepsis-3 onset timestamp	Yes under Sepsis-3 implementation	Yes under Sepsis-3	No	NR as explicit exclusion	Serial ICU event detection; 12-h lead reported	Abstract/Methods, PDF pp.1-2
Chen et al. [27]	Sepsis-3 within 7 d after liver transplantation	Yes	Yes; Delta SOFA >=2 consequent to infection	No	Yes	Daily SOFA manually checked in EMR	Methods, PDF p.3

Interpretive note. The six primary sepsis-related studies were therefore not treated as evaluations of an identical clinical endpoint. Three consistently used Sepsis-3 criteria; Shu et al. used different ascertainment approaches across development and external validation; Bunn et al. used an older ACS-NSQIP/SIRS-based definition; and Fransvea et al. modeled qSOFA >=2 as a sepsis-risk proxy.
